# A 13-gene prognostic model developed using machine learning to predict the response to neoadjuvant chemoradiotherapy in rectal carcinoma

**DOI:** 10.1186/s12935-026-04256-9

**Published:** 2026-03-21

**Authors:** Zhanhua Gao, Minghan Qiu, Zhen Yang, Xinyue Fang, Guoxing Yin, Qiaonan Zhang, Jinpu Liu, Ruxue Liu, Yayun Wang, Yuya Liu, Meng Zhang, Haiyang Zhang, Xiangqian Zheng, Hui Wang, Jie Hao, Ming Gao

**Affiliations:** 1 Department of Thyroid and Breast Surgery, Tianjin Union Medical Center, The First Affiliated Hospital of Nankai University, Tianjin, 300121 China; 2 Department of Oncology, Tianjin Union Medical Center, The First Affiliated Hospital of Nankai University, Tianjin, 300121 China; 3 Tianjin Cancer Institute of Integrative Traditional Chinese and Western Medicine, Tianjin Union Medical Center, The First Affiliated Hospital of Nankai University, Tianjin, 300121 China; 4 School of Medicine, Nankai University, Tianjin, 300071 China; 5 Department of Cell Biology and Genetics, Nankai University, Tianjin, 300071 China; 6 College of Integrative Medicine, Tianjin University of Traditional Chinese Medicine, Tianjin, 300121 China; 7 Department of Colorectal Surgery, Tianjin Union Medical Center, Tianjin Institute of Coloproctology, Nankai University, Tianjin, China; 8 Department of Thyroid and Neck Tumor, Key Laboratory of Cancer Prevention and Therapy, Tianjin Medical University Cancer Institute & Hospital, National Clinical Research Center for Cancer, Tianjin’s Clinical Research Center for Cancer, Tianjin, 300060 China

**Keywords:** Rectal carcinoma, Neoadjuvant chemoradiotherapy, Radiotherapy, Machine learning, Prognostic model

## Abstract

**Backgrounds:**

Neoadjuvant chemoradiation (nCRT) is a standard treatment for rectal carcinoma that reduces tumor size and local recurrence while improving the rate of sphincter preservation. However, many patients remain insensitive to nCRT, with some exhibiting tumor progression. To date, there is still a lack of clinically available prognostic models to differentiate the sensitivity of patients with rectal carcinoma to nCRT. This study aimed to develop a genetic predictive model that predicts the effectiveness of nCRT in patients with rectal carcinoma, offering guidance for future treatments and studies on the underlying mechanisms.

**Methods:**

Based on the NCBI GEO database datasets, key hub genes affecting the efficacy of neoadjuvant chemoradiotherapy in rectal carcinoma were identified using WCGNA. Subsequently, a consistency analysis of 101 model combinations constructed using ten different machine learning algorithms was performed in two independent cohorts, and a prognostic model (chemoradiation resistance score, CRTR score) was developed and validated. Moreover, the clinical applicability of CRTR in immunotherapy and drug selection was investigated using multi-omics analysis and public databases. Finally, the effect of KIF14 on the radiosensitivity of rectal carcinoma cells was studied using in vitro experiments.

**Results:**

The CRTR model was composed of 13 genes impacting nCRT sensitivity, whereas six genes (KIF4, DBF4, UBL4A, SLC10A3, PRRG4, and PAPSS2) acted as protective factors and seven (BMS1, DSC2, PROM2, MNAT1, PPID, SMPDL3B, and TNFRSF14) served as risk factors. The CRTR model showed a significant negative correlation with the prognosis of patients with rectal carcinoma undergoing nCRT. Furthermore, patients with higher CRTR values displayed an increased potential to benefit from immunotherapy. Drug sensitivity analysis indicated that aurora kinase inhibitors, telomerase inhibitors, JAK1 inhibitors, and others may enhance the efficacy of nCRT. Finally, we identified KIF14 as the gene that contributed the most to the model and performed preliminary validation. Radiation significantly upregulated the expression of KIF14, and overexpression of KIF14 increased the radiosensitivity of rectal carcinoma cells.

**Conclusion:**

The CRTR score, based on 13 genes, was able to predict the prognosis of patients with rectal carcinoma undergoing neoadjuvant chemoradiotherapy and demonstrated immense potential in providing personalized risk assessments and recommendations for targeted immunotherapy. The core gene, KIF14, in the CRTR model may serve as a potential predictive biomarker of radiosensitivity in rectal carcinioma.

**Supplementary Information:**

The online version contains supplementary material available at 10.1186/s12935-026-04256-9.

## Introduction

Rectal carcinoma (RC) is one of the most common malignancies in the world. According to global cancer epidemiology statistics from 2020, CRC ranks third in incidence and second in mortality among all cancers, with a steadily increasing trend [1, 2]. Currently, the standard treatment for locally advanced rectal carcinoma involves neoadjuvant concurrent chemoradiotherapy (nCRT) along with total mesorectal excision (TME) [3]. Numerous studies have confirmed that nCRT can effectively reduce tumor staging, improve the rate of R0 resections, and decrease the rate of local recurrence [2, 4]. However, the patient responses to nCRT varied significantly. While 40%-70% of patients may achieve tumor remission following nCRT, a subset remains insensitive, potentially delaying surgical interventions due to tumor progression [5, 6]. Thus, the precise prediction of the response to nCRT and selection of suitable patients for this treatment is a continuing challenge in rectal carcinoma therapy.

Biomarkers based on genetic phenotypes have been extensively utilized to assist in the selection of oncological treatment regimens and forecasting outcomes. For instance, KRAS and BRAF mutations direct the use of targeted therapies in metastatic rectal carcinoma [7, 8], while microsatellite stability affects immunotherapy choices [9]. However, effective biomarkers for guiding nCRT are still lacking owing to inadequate statistical methods, lack of validation, and poor data utilization in model development [10]. Moreover, the complex interplay between genes requires thorough analysis to uncover the underlying mechanisms [11–13], highlighting the need for broader genetic studies in rectal carcinoma.

**Fig. 1:**
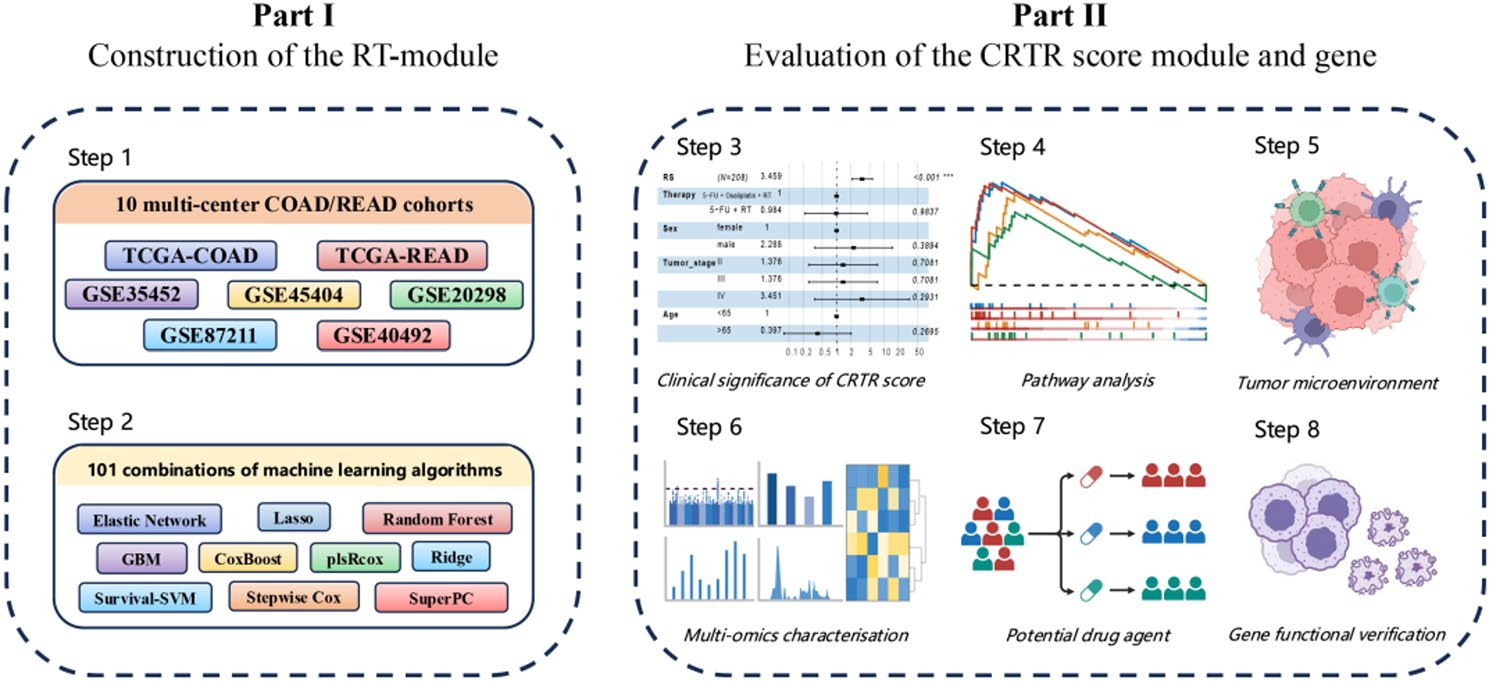
The overall design of this study

In this study, we used multiple datasets to identify genes related to the sensitivity of CRC to chemoradiotherapy and combined ten machine learning algorithms in various model parameters to develop and validate a chemoradiotherapy resistance prediction score (CRTR Score) for nCRT in rectal carcinoma patients (Fig. 1). The 13-gene CRTR score could accurately predict the outcomes of nCRT. Preliminary in vitro studies suggested that KIF14, a key gene in the CRTR model, is functionally associated with radiosensitivity in colorectal cancer. This study aimed to optimize treatment and improve outcomes in patients with rectal carcinoma.

## Methods and materials

### Data collection and processing

We obtained independent public datasets from The Cancer Genome Atlas (TCGA) (https://portal.gdc.cancer.gov/) and the NCBI GEO database (https://www.ncbi.nlm.nih.gov/).

Datasets used for identifying chemoradiation sensitivity genes in RC: (1) GSE35452 [14] and GSE45404 [15]: Contain data on tumor shrinkage (TRG) from patients treated with preoperative nCRT, all treated with 50.4 Gy/28F synchronous fluorouracil chemotherapy, where 41 patients were identified as sensitive (response) and 49 patients as resistant (non-Response) to nCRT; GSE20298 [16]: data on the survival of 12 colorectal cancer cell lines following exposure to 3µM 5-fluorouracil and 2 Gy radiation.

Datasets utilized to build and validate machine learning models for nCRT prognosis prediction: (1) GSE40492 and GSE87211 [17]: 245 and 203 patients, respectively, who underwent nCRT with full data on overall survival (OS) and relapse-free survival (RFS); (2) GSE60331 [18] comprised 7 patients who responded effectively to nCRT and 9 who did not (Response: TRG 0–1, Non-response: TRG 2–3); and (4) TCGA-COAD and TCGA-READ: for conducting multi-omics analysis of CRTR scores in colorectal cancer.

Affymetrix platform data (GSE35452, GSE45404, and GSE60331) were processed using the Robust Multi-array Average (RMA) method using the Affy package. Agilent platform data (GSE40492 and GSE87211) were used for array signal extraction, background adjustment, and normalization using the limma package. The META-GEO cohort was adjusted for batch effects using the ComBat package, merging the GSE35452 and GSE45404 datasets from the Affymetrix GPL570 platform. For the TCGA datasets, raw RNA-seq counts were transformed into TPM. Somatic mutations were processed following TCGA MuTect2 protocol and analyzed using the maftools package, with somatic copy number variations evaluated using GISTIC2.0.

### Weighted Gene Co-expression Network Analysis (WGCNA)

Co-expression networks for the META-GEO and GSE20298 datasets were constructed using the WGCNA package [19]. To maintain the characteristics of a scale-free network and low average connectivity, we selected R2 > 0.9 as the threshold and adjusted β to achieve an average connectivity close to 0. Specifically, for GSE20298, we set the minimum module size to 60 and the module merging threshold to 0.5, clustering genes with high similarity into 13 color-coded modules by using dynamic tree cutting. Module characteristic genes were selected based on MM > 0.6 and |GS| > 0.3. For GSE35452 and GSE45404, we set the minimum module size to 60 and the module merging threshold to 0.25, clustering the genes into 16 color-coded modules using dynamic tree cutting. Module characteristic genes were selected based on MM > 0.5 criteria. The gray module consisted of genes that could not be classified into clusters.

### Machine learning-based nCRT prognostic gene signature

For the development of the CRTR genetic model predicting nCRT outcomes, we used 10 machine learning algorithms and 101 combinations based on Liu et al. [20]. Comprehensive algorithms include Random Survival Forests (RSF), Elastic Net (Enet), Lasso, Ridge, Stepwise Cox, CoxBoost, Cox Partial Least Squares Regression (plsRcox), Supervised Principal Component (SuperPC), Generalized Boosting Regression Model (GBM), and Survival Support Vector Machine (survival-SVM). The generation process was as follows: (1) In the GSE40492 cohort, 101 algorithm combinations were applied to chemoradiotherapy sensitivity-related genes, with predictive models fitted using a Leave-One-Out Cross-Validation (LOOCV) framework; (2) these models were validated in the GSE87211 dataset; (3) For each model, Harrell’s Consistency Index (C-index) was calculated across all datasets, and the model with the highest average C-index was considered the best model. Using this model, each sample in the training and validation cohorts was scored, resulting in a prognostic score predictive of outcomes for colorectal cancer patients treated with nCRT (CRTR score).

### Assessing the predictive value and potential clinical applications of CRTR score

The samples were categorized into two groups based on their CRTR scores. The prognostic efficacy of CRTR was evaluated using Kaplan-Meier survival analysis and receiver operating characteristic curves (ROC). To increase the clinical utility of CRTR, clinical characteristics were integrated with CRTR scores to construct predictive nomograms using multivariable Cox regression, providing individualized probability outcomes based on personal attributes. Time-dependent ROC curves were employed to validate the accuracy of the model, and decision curve analysis was used to evaluate the clinical benefits for patients.

### Biological function and pathway enrichment analysis

We used selection criteria of FDR < 0.05, log2(FC) > 1, and applied the limma package to identify genes significantly differentially expressed between the high CRTR and low CRTR groups. To explore the biological functions and pathways associated with CRTR, we conducted Gene Ontology (GO) and Kyoto Encyclopedia of Genes and Genomes (KEGG) analyses using the clusterProfiler package [21]. Briefly, the differentially expressed genes were converted to Entrez IDs for input into GO and KEGG enrichment analyses with an adjusted p-value threshold of < 0.05. Finally, Gene Set Enrichment Analysis (GSEA) was performed to assess the differential genes between the two CRTR groups.

### Assessment of the immune microenvironment and response to immunotherapy

We used the IOBR R package, including CIBECRTRORT, CIBECRTRORT-ABS, QUANTISEQ, MCP-counter, Xcell, TIMER, and EPIC, to estimate the relative infiltration of various immune cell subpopulations. Preliminary analysis of TCGA somatic mutation data was conducted using the maftools R package to calculate and compare TMB differences between groups. The tumor immune dysfunction and exclusion (TIDE) (http://tide.dfci.harvard.edu/) algorithm was employed to predict the sensitivity of high and low CRTR groups to immune checkpoint inhibitors.

### Selection of therapeutic drugs related to CRTR score

Expression data for human tumor cell lines were obtained from the Broad Institute’s Cancer Cell Line Encyclopedia (CCLE, https://portals.broadinstitute.org/ccle/about/). Data on the drug sensitivity of CCLs were obtained from the Cancer Therapeutics Response Portal (CTRP2, https://portals.broadinstitute.org/ctrp) and the Genomics of Drug Sensitivity in Cancer (GDSC2, https://www.cancerrxgene.org/). The area under the dose-response curve (AUC) and half-maximal inhibitory concentration (IC50) were used as measures of drug sensitivity for CTRP2 and GDSC2, respectively. The oncoPredict package was used to train models on the CTRP2 and GDSC2 datasets using ridge regression, and these models were applied to the GSE40492 and TCGA-CO/READ datasets to predict the drug sensitivity for each patient. Lower AUC/IC50 values are associated with higher drug sensitivity. The Spearman correlation between the AUC/IC50 values and CRTR was calculated, and drugs with a negative correlation were considered beneficial for the high CRTR group.

### Establishment of stable cell lines

The KIF14 lentiviral overexpression plasmid was purchased from HedgeHogbio (Shanghai, China). 293FT cells were cultured to 70%-80% confluence. The target KIF14 plasmid, vector plasmid, and packaging plasmids (psPAX2 and pMD2.G) were mixed in 500 µL of Opti-MEM medium and added to 293 T cells. The viral supernatant was collected and added to CACO-2 cells with 10 µg/mL polybrene. Both the virus and control cells were treated with 1 µg/mL puromycin. Cells surviving in the virus group were deemed to be successfully infected.

### Western blotting

The cells were lysed to extract cellular proteins. The extracted proteins were denatured and separated by SDS-PAGE based on their molecular weights. After electrophoresis, the proteins were transferred onto a PVDF (polyvinylidene fluoride) membrane using a gel-matrix sandwich setup. The PVDF membrane was then incubated sequentially with primary and secondary antibodies, followed by exposure to the developing solution to obtain images of the target bands.

### Cell Counting Kit-8 (CCK-8) assay

The cells were seeded in 96-well plates at a density of 1000 cells/well. At 0, 24, 72, 120, and 168 h, 100 µL of 10-fold diluted CCK-8 reagent was added and the cells were incubated at 37 °C with 5% CO2 for 2 h. absorbance at OD450 was then measured using a microplate reader.

### Cell irradiation and clonogenic assay

Cells were seeded into six-well plates at densities of 250, 800, 1000, 10,000, and 30,000 cells per well, cultured for eight hours (overnight) until they adhered, and then irradiated. After adhesion, the cells received varying doses of X-rays (0, 2, 4, 6, and 8 Gy). Two weeks later, cells were fixed with 4% paraformaldehyde and stained with crystal violet. The experiment was independently repeated thrice. Colonies containing 50 or more cells were counted.

### Statistical analyses

Data analysis and visualization were conducted using R software (version 4.3.1). All experiments were conducted at least three times, and the results are expressed as mean ± standard deviation. Spearman’s test was used to assess correlations between continuous variables. Categorical variables were compared using the chi-square test, whereas continuous variables were compared using the Wilcoxon rank-sum test or t-test. A P-value less than 0.05 was considered statistically significant (*P < 0.05, **P < 0.01, ***P < 0.001, ns: not significant).

## Result

### Identification of key genes affecting rectal cancer neoadjuvant chemoradiotherapy efficacy using WGCNA

**Fig. 2:**
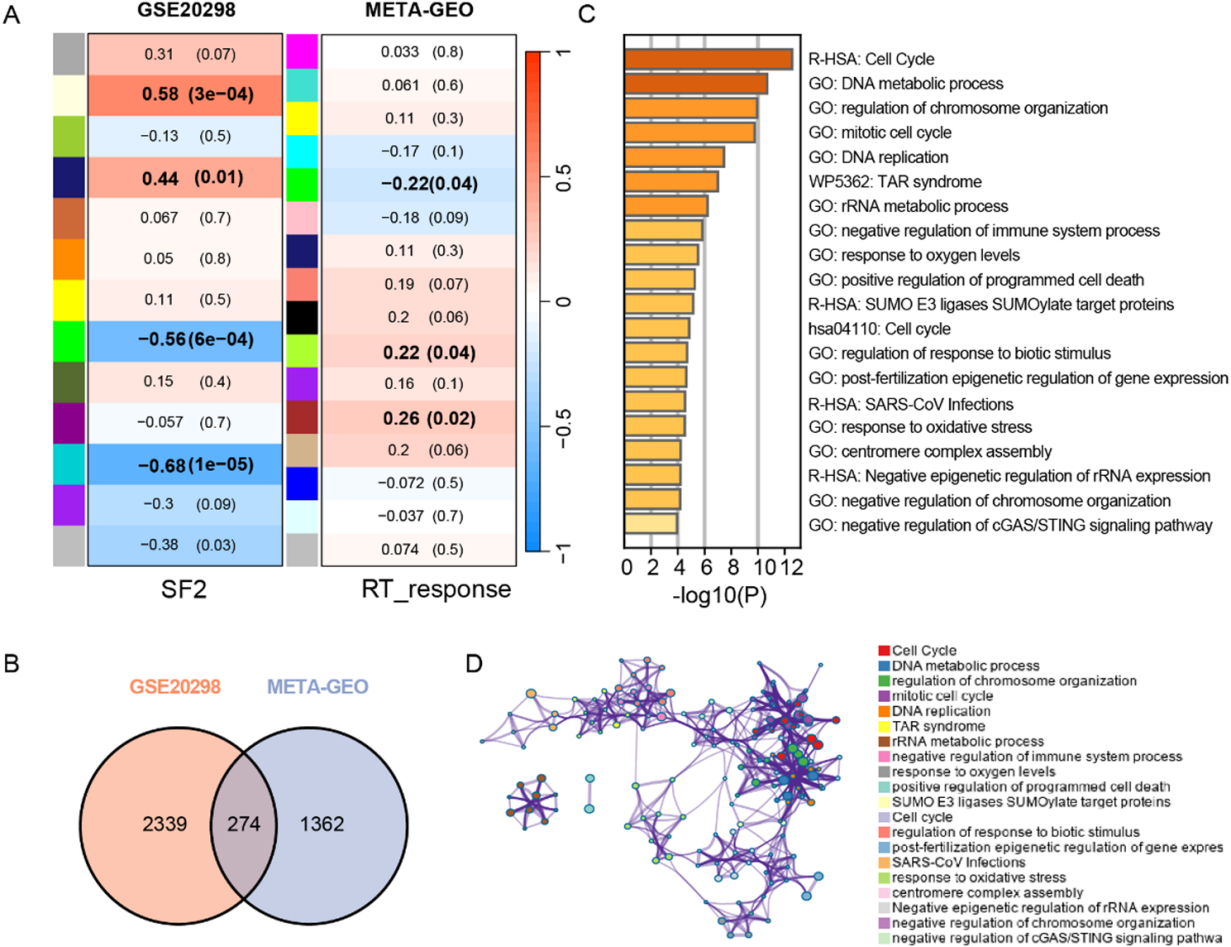
Identification of chemoradiotherapy sensitivity-related genes via WGCNA in the GSE20298 dataset of colorectal cancer cells and the META-GEO dataset of rectal cancer patients. **(A)** The heatmap revealing the relationship between module eigengenes and SF2 or response to chemoradiotherapy. The correlation (upper) and p-value (bottom) of module eigengenes and SF2 or response to chemoradiotherapy were presented. Modules with a p-value less than 0.05 were defined as chemoradiotherapy sensitivity-related modules. **(B)** A total of 274 chemoradiotherapy sensitivity-related genes were identified by taking the intersection between GSE20298 key modules genes and META-GEO key modules genes via the venn diagram. **(C)** Enrichment in the pathways of chemoradiotherapy sensitivity-related genes. The color indicates significance of the enrichment terms. **(D)** Enrichment analysis of interaction network by Metascape for chemoradiotherapy sensitivity-related genes. Network of enriched terms colored by cluster identity, where nodes that share the same cluster identity are typically close to each other

The response to neoadjuvant chemoradiotherapy significantly varies among patients with rectal cancer. To identify the key genes affecting treatment efficacy, the GSE20298 dataset, which includes chemoradiotherapy sensitivity data for colorectal cancer cell lines, was obtained from the GEO database. To further screen for gene sets that may influence the efficacy of neoadjuvant chemoradiotherapy in rectal cancer, we searched the NCBI GEO database for available rectal cancer neoadjuvant chemoradiotherapy TRG-related datasets. The GSE35452 and GSE45404 datasets were selected, which used consistent nCRT protocols (50.4 Gy/28F + 5-FU ± LV) and the same sequencing platform (GPL570). These data were named META-GEO after batch-effect normalization.

The WGCNA algorithm was used to analyze the correlation between gene modules and SF2 (the survival fraction of tumor cells after 2 Gy irradiation and 3µM 5-FU treatment) within the GSE20298 group as well as the correlation between gene modules and nCRT response (response or non-response) within the META-GEO group. Soft thresholds β were set to 13 and 3, respectively. Heatmaps were produced to show the correlation between the gene modules and radiotherapy sensitivity (Fig. 2A, Supplementary Fig. 1C-D).

We selected genes with statistical significance (*P* < 0.05) from the modules for further analysis (bold in Fig. 2A). In the GSE20298 group, 2613 hub genes were identified from four significant modules, and 1636 hub genes were identified from two modules in the META-GEO group. By intersecting the two sets, 274 hub genes related to nCRT sensitivity in rectal cancer were identified (Fig. 2B). Enrichment analysis of these genes was performed. GO enrichment revealed that key nCRT genes were mainly enriched in processes such as the cell cycle, DNA metabolism, chromosome organization, and DNA replication. KEGG analysis showed enrichment in pathways such as the cell cycle, DNA metabolism, chromosome regulation, and oxidative stress response (Fig. 2C-D), which are critical for chemoradiotherapy sensitivity, aligning with known sensitization/resistance mechanisms.

### The 13-gene model accurately predicts the prognosis of rectal cancer patients undergoing nCRT

**Fig. 3:**
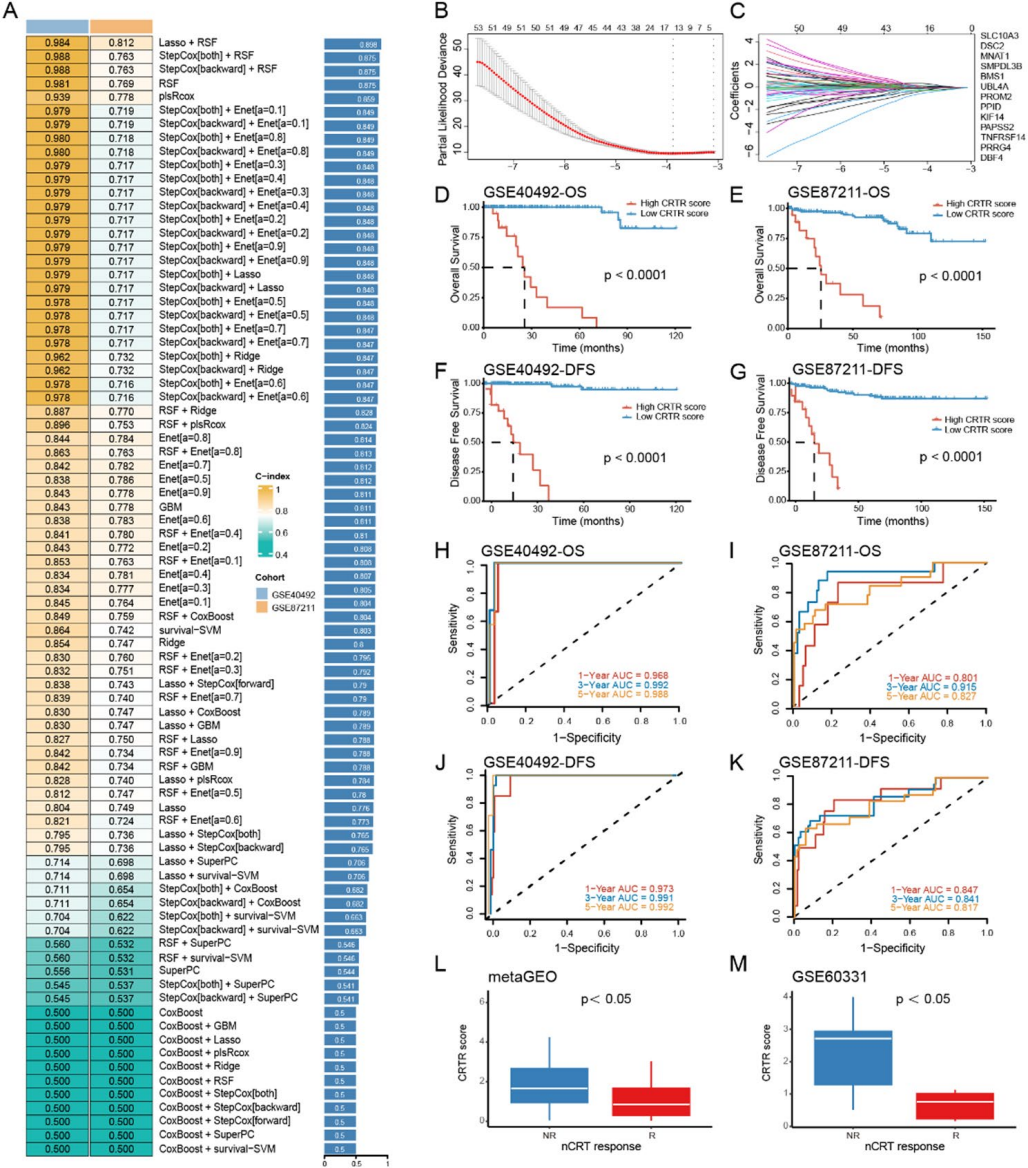
Construction and validation of CRTR score based on machine learning. **A.** Through a comprehensive computational framework, a combination of 87 machine learning algorithms was generated. The C-index of each model was calculated through the GSE40492 and GSE87211 cohorts and sorted by the average C-index of each set. **B-C.** In the GSE40492 cohort (*n* = 245), the determination of the optimal λ was obtained when the partial likelihood deviance reached the minimum value, and further generated Lasso coefficients of the most useful prognostic genes. **D-G.** Kaplan–Meier curves of OS and DFS according to the CRTR score in GSE40492 and GSE87211 cohorts. **H-K.** Receiver operating characteristic (ROC) curves showing 1-, 3-, and 5-year OS and DFS in GSE40492 and GSE87211 cohorts. **L-M.** The distribution of CRTR score between responders and nonresponders of nCRT in META-GEO and GSE60331 cohorts; Response: TRG 0–1, Non-response: TRG 2–3

To predict rectal cancer nCRT efficacy, we used 10 algorithms and 101 parameter combinations in machine learning to select the optimal model based on 274 hub genes. GSE40492 served as the training set and GSE87211 was used as the validation set. Among the 101 parameter combinations, 82 successfully completed the model construction. The results showed that the Lasso-RSF framework achieved the highest CI value (Fig. 3A). This model included the 13 most effective feature genes: KIF14, DBF4, UBL4A, SLC10A3, PRRG4, PAPSS2BMS1, DSC2, PROM2, MNAT1, PPID, SMPDL3B, and TNFRSF14 (Fig. 3B-C). We calculated scores for each patient based on this framework.

Prognostic analysis showed that a high score was associated with lower overall survival (OS) and disease-free survival (DFS) (Fig. 3D-G). The score demonstrated excellent predictive performance for both OS and DFS in both the training and validation sets: In the GSE40492 training set, the AUC values for predicting 1-year, 3-year, and 5-year OS were 0.968, 0.992, and 0.988, respectively, while in the GSE87211 validation set, the AUC values were 0.801, 0.915, and 0.827. For DFS, the AUC values in GSE40492 were 0.973, 0.991, and 0.992 for the 1-year, 3-year, and 5-year predictions, respectively, while in GSE87211 the values were 0.847, 0.841, and 0.817 (Fig. 3H-K).

Moreover, in the META-GEO dataset, the score was notably higher in patients who responded poorly to preoperative concurrent chemoradiotherapy (Fig. 3L). The same trend was observed for the GSE60331 dataset (Fig. 3M). Furthermore, in the LOVO and HCT-116 radiotherapy-resistant cell lines previously constructed and published by our research group [22], we found that radiotherapy-resistant cells had higher CRTR scores (Supplementary Fig. 1G). These findings confirm the strong prognostic power of the 13-gene CRTR score for patients with rectal cancer undergoing nCRT, with higher scores linked to worse outcomes. Therefore, we named this score the Chemoradiotherapy resistance prediction score (CRTR Score).

### Nomogram model to predict the prognosis of rectal cancer after nCRT using CRTR scores

**Fig. 4:**
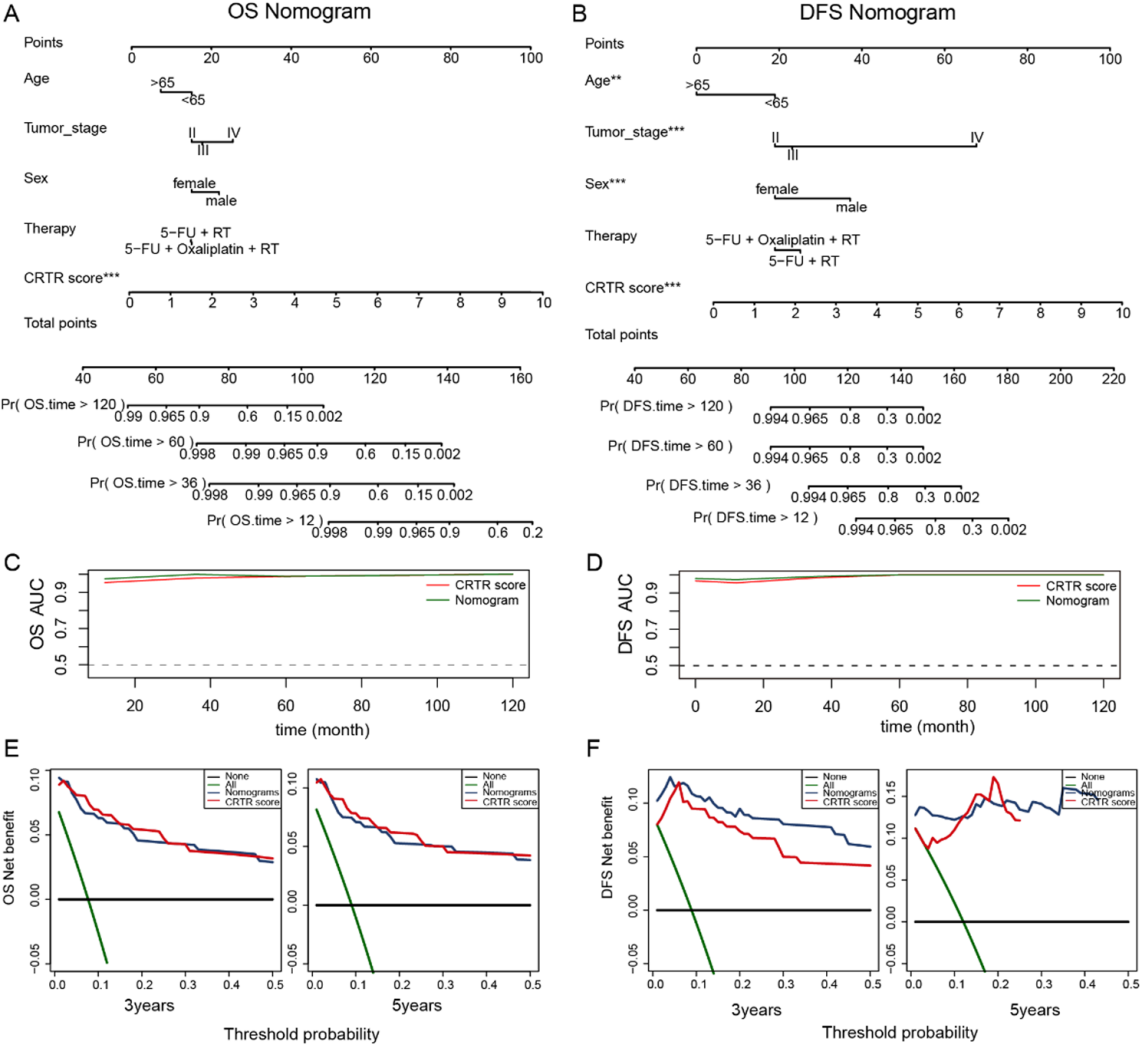
Building and validating nomograms.** A-B.** A comprehensive nomogram was constructed based on CRTR score and the clinical parameters to predict 1-, 3-, 5-, and 10-year OS and DFS in GSE40492 cohort. **C-D.** Comparison of the time-dependent ROC curves between the comprehensive nomogram and CRTR score. **E-F.** DCA curves were compared over 3-year and 5-year periods in terms of OS and DFS for patients. The x-axis indicates the threshold probability, and the y-axis represents the net benefit

To evaluate the clinical predictive validity of the CRTR score more effectively, we included patient clinical characteristics and, using multivariable Cox regression, built a nomogram model based on GSE40492 around the CRTR score model to estimate OS and DFS. Compared to these clinical factors, the CRTR score contributed the most to both nomogram prediction models (Fig. 4A-B).

Time-dependent ROC curve analysis demonstrated that the OS and DFS nomogram prediction models possessed excellent predictive values (AUC > 0.9), and the AUC of the CRTR score was similar to that of the nomogram models (Fig. 4C-D). The DCA curve revealed that both nomogram prediction models exhibited significant net benefits at 3 and 5 years, respectively, with the CRTR score curve aligned most closely with the model curves and far exceeded other clinical prognostic factors (Fig. 4E-F). These results collectively indicate that the CRTR score, as an independent prognostic factor, offers a substantial advantage in predicting the prognosis of patients with rectal cancer treated with neoadjuvant chemoradiotherapy.

### Annotation of biological mechanisms related to CRTR score

**Fig. 5:**
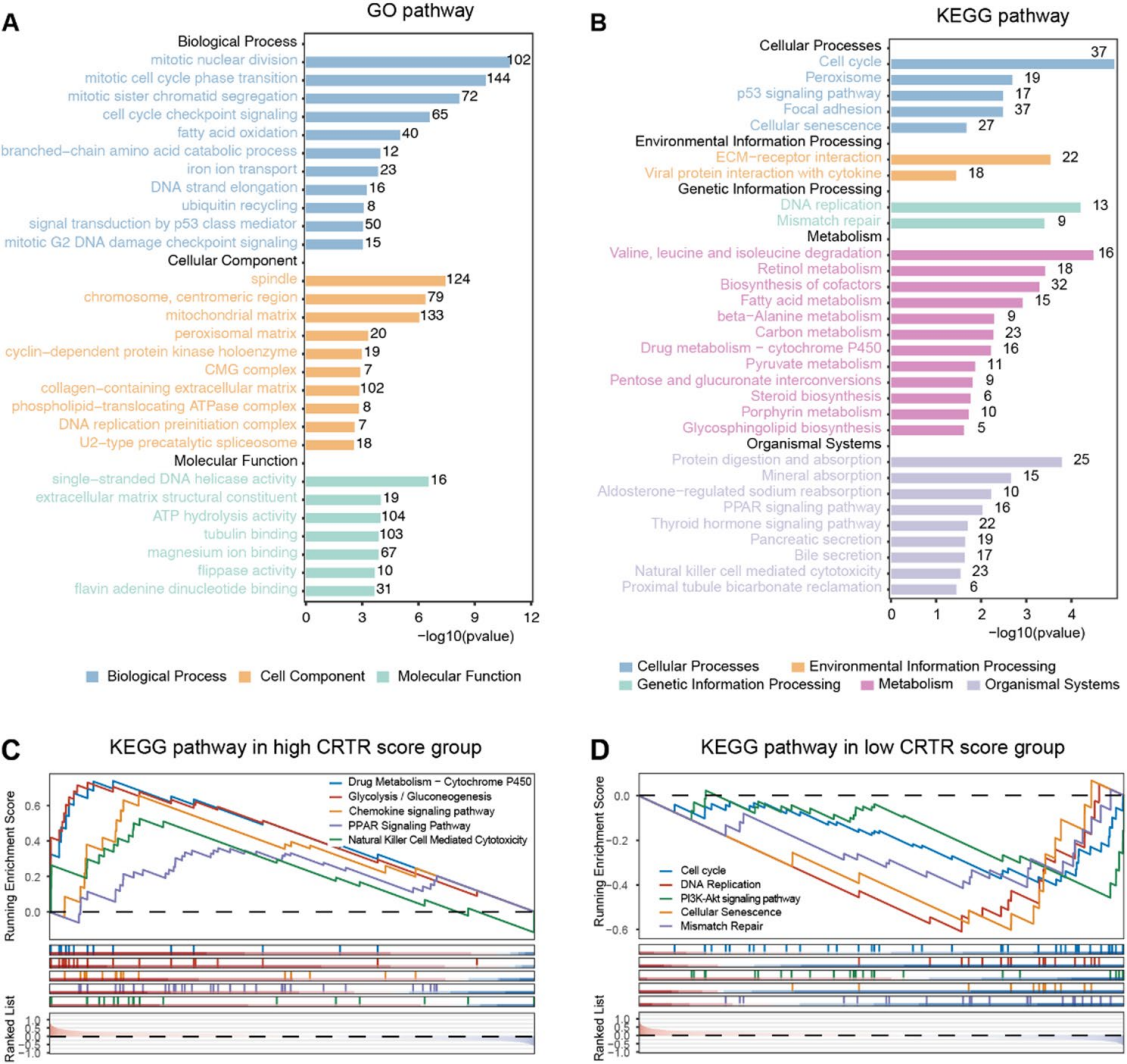
Functional enrichment analysis of CRTR signature.** A-B.** GO and KEGG enrichment analysis of DEGs between high- and low-CRTR group. **C-D.** The GSEA enriched pathways in the high-CRTR group and low-CRTR group

Using CRTR scores, we split the GSE40492 cohort at the median and identified DEGs between the high and low CRTR groups. GO analysis (Fig. 5A) indicated that the enriched genes were mainly associated with cell cycle regulation, DNA repair, mitosis-related biological processes, and cellular components. KEGG pathway analysis (Fig. 5B) revealed that DEGs were enriched in cell cycle, p53 signaling, drug metabolism, and immune response pathways. In the GSEA analysis, a high CRTR score was correlated with increased activity in drug metabolism (e.g., cytochrome P450), gluconeogenesis, and immune response (e.g., natural killer cell-mediated cytotoxicity) pathways (Fig. 5C). In contrast, a low CRTR score was linked to a more active cell cycle, DNA replication, and PI3K/AKT signaling pathways (Fig. 5D). These findings underscore the significance of the cell cycle, DNA damage repair, drug metabolism, and immune response in modulating rectal cancer sensitivity to chemoradiation, suggesting potential new targets for future treatment strategies.

### Comparison of multi-omic and immune characteristics between two mtPCDI groups

**Fig. 6:**
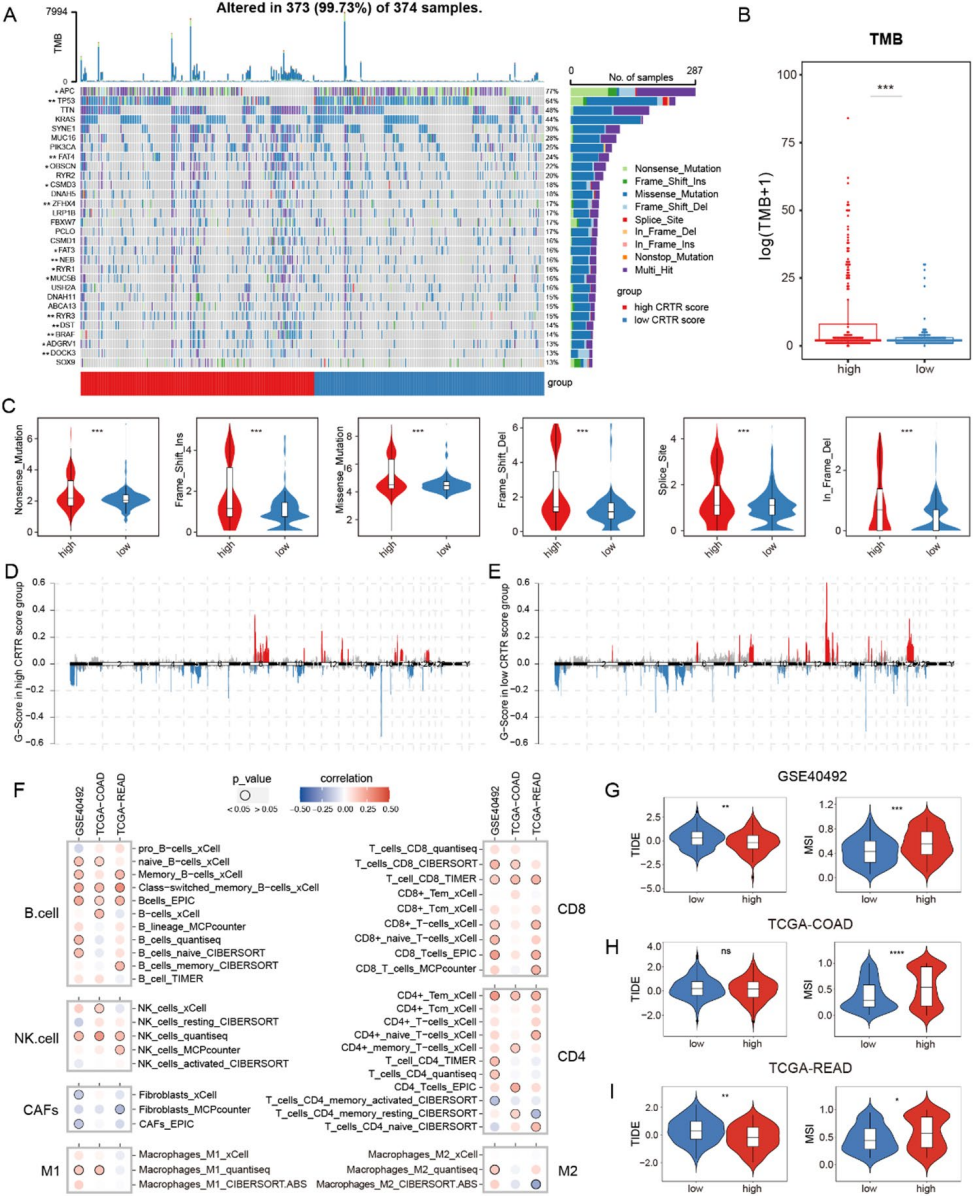
Multi-omic and immune characteristics.** (A)** The waterfall plot of the top 30 somatic gene mutations, with corresponding proportions of mutations in each group depicted in the bar plot on the right. **(B)** Comparison of high- and low- CRTR patients of TMB in the TCGA-COADREAD dataset. **(C)** Comparison of nonsense mutation, frameshift insertion, missense mutation, frameshift deletion, splice site and inframe deletion between high- and low-CRTR groups. **D-E.** Visualization of patients’ copy number amplification(red) and deletion(blue) using chromosomal plots in the high- and low- RS groups. **F.** Correlation of CRTR with immune cell infiltration evaluated using seven algorithms (TIMER, EPIC, xCELL, CIBERSORT, CIBERSORT-ABS, QUANTISEQ and MCPcount) across GSE40492, TCGA-COAD and TCGA-READ cohorts. **G-I.** Violin plot comparing tumor immune dysfunction and exclusion (TIDE) scores and microsatellite instability (MSI) between the low- and high- CRTR groups

Using TCGA-COADREAD data, we identified 15 genes, including APC, TP53, and BRAF, with significantly different mutation frequencies between the high and low CRTR score groups (Fig. 6A). The high CRTR score group had higher TMB levels (*P* = 0.0035) (Fig. 6B) and more frequent mutation patterns (Fig. 6C). Chromosome mutation analysis revealed more 8q amplifications in the high CRTR group and more 13q and 20q amplifications in the lower group (Fig. 6D-E). Additionally, patients with high CRTR scores showed more co-occurring mutations, indicating a poorer radiotherapy response and prognosis (Supplementary Fig. 3).

Subsequently, we used seven algorithms to assess immune cell infiltration and found that the CRTR score was positively correlated with CD8 + T cells, B cells, NK cells, and M1 macrophages, while it was negatively correlated with CAFs and M2 macrophages (Fig. 6F). Using the Tumor Immune Dysfunction and Exclusion (TIDE) tool, we found that patients with high CRTR scores had significantly lower TIDE scores, higher Microsatellite Instability (MSI) score (Fig. 6G-I), suggesting that patients with high CRTR scores may benefit more from immunotherapy.

### Drug sensitivity analysis based on the CRTR score model

**Fig. 7:**
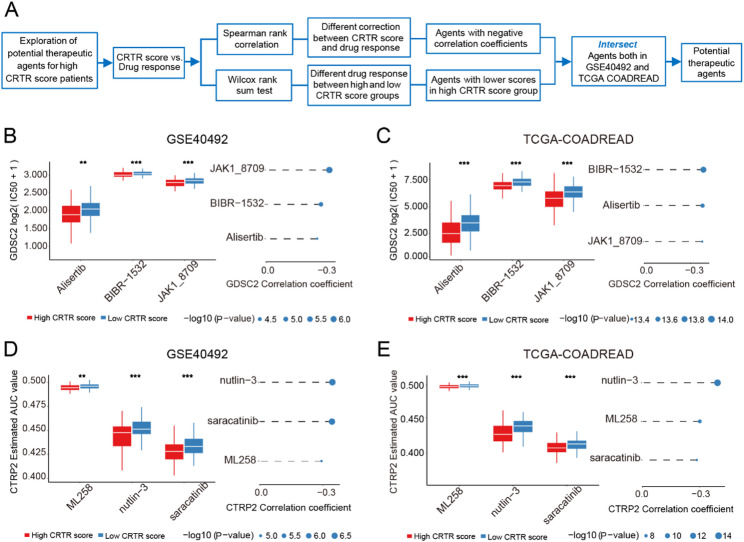
Evaluating therapeutic drug benefits. **A.** Schematic outlining the strategy to develop potential therapeutic agents with higher drug sensitivity in the high CRTR group. **B-E.** The correlation and differential analysis of drug sensitivity for potential drugs screened from GDSC2 and CTRP2 datasets in GSE40492 and TCGA-COADREAD, the lower values on the y-axis of boxplots imply greater drug sensitivity

To identify potential drugs that could enhance the efficacy of nCRT, we analyzed the correlation between the CRTR score and response to various drugs. The analysis process is illustrated in Fig. 7A. Using data from the GDSC2 and CTRP2 databases, we identified several drugs whose AUC or IC50 values positively correlated with the score: In the GDSC2 database, we identified Aurora A kinase inhibitor Alisertib, telomerase inhibitor BIBR-1532, and JAK1 inhibitor JAK1-8709 (Fig. 7B-C); in the CTRP2 database, we found p53 inhibitor nutlin-3, Src inhibitor saracatinib, and protease inhibitor ML258 (Fig. 7D-E). Patients with high CRTR scores may exhibit better sensitivity to these agents, suggesting that these drugs could improve the outcomes of nCRT in rectal cancer.

### KIF14 can increase the radiation sensitivity of colorectal cancer cells

**Fig. 8:**
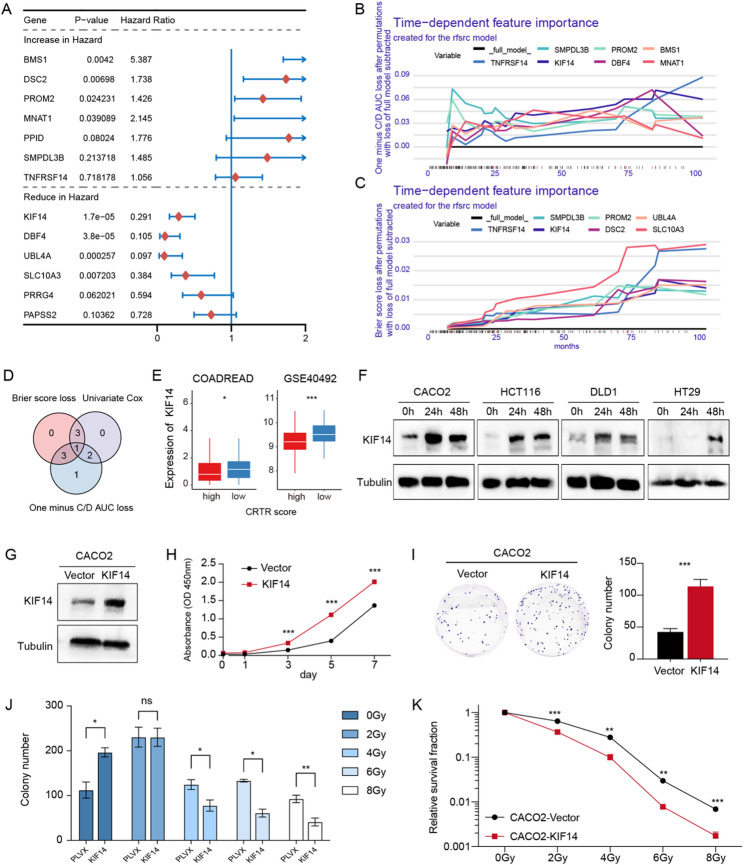
The effect of KIF14 overexpressing on radiotherapy response in colorectal cancer cell lines.** A.** The univariate Cox regression analysis results of 13 model genes in GSE40492 cohort. **B-C.** Changes in the importance of time-dependent feature. **D.** The Venn diagram illustrates the overlap between the significant genes identified in the HR forest plot (*P* < 0.01) and the genes with substantial contributions to the CRTR model. **E.** The differential expression analyses of KIF14 in low- and high- CRTR groups. **F.** Western blot analysis of KIF14 expression at 0 h, 24 h and 48 h after irradiation in colorectal cancer cell lines(CACO-2, HCT-116, DLD-1 and HT-29). **G.** Identification of stably transfected cells overexpressing exogenous KIF14. **H-I.** CCK-8 and plate cloning assay to detect the proliferation of ability of CACO-2 cells after overexpression of KIF14. **J-K.** CACO2-Vector and CACO2-KIF14 cells were used for clonogenic formation assay, and the results were displayed through the histogram and a linear quadratic model. The experiment was repeated three times independently

To identify potential nCRT sensitivity targets in colorectal cancer, we conducted a univariate COX regression analysis of the above 13 genes in the CRTR score model using the GSE40492 cohort. Eight genes were significantly associated with patient survival: BMS1, DSC2, PROM2, and MNAT1 were associated with worse prognosis, while KIF14, DBF4, UBL4A, and SLC10A3 were associated with better outcomes (Fig. 8A).

Additionally, we assessed the CRTR model using time-dependent metrics, such as C/D AUC curves and Brier score, to identify key variables. Four genes, SMPDL3B, KIF14, PROM2, and TNFRSF14, were identified as major contributors (Fig. 8B-C). Among them, KIF14 showed the strongest mortality risk correlation (*p* = 1.7 × 10 − 5) and had a lower Brier score with a higher and more stable C/D AUC (Fig. 8D). Its expression was significantly higher in the low CRTR score group across both the TCGA-COADREAD and GSE40492 cohorts (Fig. 8E).

To further validate the role of KIF14 in CRC, we irradiated four human CRC cell lines (CACO-2, HCT116, DLD-1, and HT29) with 4 Gy. Western Blot results showed increased KIF14 expression post-radiation in all cell lines, especially in CACO-2 (Fig. 8F), suggesting KIF14’s involvement in CRC radiotherapy response. Subsequently, we overexpressed KIF14 gene in CACO-2, HT29 and DLD1 cells (Fig. 8G, Supplementary Fig. 6A). The experiment revealed that overexpression of KIF14 enhanced the proliferation rate and colony-forming ability of CRC cells (Fig. 8H-I, Supplementary Fig. 6B). In combination with radiation, colony formation assays showed that KIF14 overexpression significantly reduced the number of colonies and decreased the survival fraction compared to control cells (Fig. 8J-K, Supplementary Fig. 6D-G). These results suggested that KIF14 significantly enhanced the radiosensitivity of rectal cancer cells.

## Discussion

In the treatment of locally advanced rectal cancer, nCRT can improve local tumor control, reduce local recurrence, and increase patient survival [2]. However, there were significant differences in patients’ responses to nCRT, reflecting the heterogeneity of rectal cancer. Therefore, identifying the sensitivity of patients with rectal cancer to nCRT is crucial for achieving personalized treatment. Despite this, there is still a lack of effective prognostic models in clinical practice to differentiate nCRT sensitivity in rectal cancer patients [23]. Current rectal cancer prognostic models typically depend on individual preferences in algorithm selection or lack validation across multiple datasets, resulting in poor model performance or overfitting [13]. In this study, we combined both cell-level and patient clinical tissue data to identify key genes related to rectal cancer chemoradiotherapy sensitivity using WGCNA. Based on these key genes, we performed batch analysis using 10 machine learning algorithms and 82 algorithm combinations, and the best prognostic model for rectal cancer (CRTR score) was obtained using the Lasso + RSF algorithm combination. The CRTR score, which relies on the expression of 13 genes, demonstrated outstanding performance across various validation datasets, offering reduced dimensionality and improved generalizability.

Sporadic colorectal cancer arises from mutations in three key genes, described by two distinct developmental sequences: (1) APC and KRAS mutations observed in the adenoma-carcinoma sequence, and (2) BRAF-V600E mutations observed in the serrated polyp-carcinoma sequence [24]. Our findings indicated that patients with high CRTR scores (resistive to nCRT) exhibited fewer APC and TP53 mutations but a higher frequency of BRAF mutations. Moreover, chromosomal mutation maps revealed that patients with lower CRTR scores (sensitive to nCRT) exhibited a higher prevalence of 20q amplification, consistent with prior studies. Previous studies have shown that Chr20q amplification is the most common copy number alteration in colorectal cancer, and MSS patients with Chr20q amplification have better recurrence-free survival than MSS patients without Chr20q amplification [25]. This further validated the effectiveness of the constructed CRTR score model.

Neoadjuvant chemoradiotherapy not only directly kills tumor cells, but also reshapes the local tumor microenvironment, enhancing the efficacy of immunotherapy [26, 27]. The consensus molecular subtypes (CMS) is a widely accepted framework for describing colorectal cancer heterogeneity [28]. We did not perform a formal CMS assignment, yet the CRTR-high group shows CMS1-like features, including higher immune cell infiltration, higher TMB, and a higher frequency of BRAF mutation. In our cohorts, CRTR-high was associated with increased CD8 + T cells and NK cells, whereas CAF infiltration was more pronounced in CRTR-low. In line with this immune contexture, TIDE predicted lower immune evasion in CRTR-high than in CRTR-low, which suggests a potential benefit from immunotherapy. These observations are hypothesis-generating computational readouts rather than clinical evidence, but together with the CMS-concordant features they support prioritizing immunotherapy or other immunotherapy-based combinations in CRTR-high.

Moreover, we discovered that patients with high CRTR scores exhibited increased sensitivity to eight common targeted therapies including BIBR1532, Alisertib, JAK1_8709, and saracatinib. BIBR1532 is a highly selective telomerase inhibitor, with telomerase enhancing the radiotherapy resistance of tumor cells by repairing damaged telomeres [29]. Alisertib is an Aurora A inhibitor that increases the sensitivity of various cancers, such as colorectal, breast, and non-small cell lung cancers, to radiotherapy [30, 31]. JAK1_8709 is a JAK1 inhibitor, and research has demonstrated that in radiotherapy-resistant colorectal cancer tissues, activation of the JAK/STAT signaling pathway restricts apoptosis and facilitates continuous cell proliferation after radiotherapy, significantly contributing to radiotherapy resistance [32]. Saracatinib is an Src kinase inhibitor. Studies have shown that the Src pathway is activated in more than 70% of colorectal tumors [33]. Inhibition of Src can significantly enhance the sensitivity of malignant glioma, esophageal, and lung cancer cells to radiotherapy by increasing DNA damage and inducing cellular senescence [34]. These drugs are potential candidates for improving the efficacy of nCRT in rectal cancer, and warrant further investigation.

The CRTR score was constructed from 13 core genes, of which six (KIF14, DBF4, UBL4A, SLC10A3, PRRG4, and PAPSS2) were protective factors and seven (BMS1, DSC2, PROM2, MNAT1, PPID, SMPDL3B, and TNFRSF14) were risk factors. We observed that the majority of these genes are linked to malignant proliferation, unfavorable prognosis, and the tumor immune microenvironment in colorectal cancer. For instance, DBF4 is a direct downstream target of the critical DNA damage repair genes ATM and ATR, and can impact cell cycle arrest following DNA damage. Our analysis identified KIF14 as the most important gene in this model. KIF14 is a microtubule-associated motor protein [35]. Research has demonstrated that KIF14 has oncogenic properties, and its high expression is linked to poor prognosis in gastric, epithelial ovarian, and hepatocellular carcinomas [36–38]. However, other studies have suggested that KIF14 may have tumor-suppressive effects in certain cancers, with its specific role possibly depending on the tumor type and environment [39, 40]. In this study, high KIF14 expression was associated with longer overall survival, which is consistent with previous findings [39]. Further experiments showed that KIF14 promoted CRC cell proliferation, but enhanced the efficacy of radiotherapy in CRC. In conclusion, our results suggest that KIF14 may serve as a potential predictive biomarker of radiosensitivity in colorectal cancer, and further studies are warranted to elucidate its underlying mechanisms.

In conclusion, the 13-gene-based CRTR score model reliably predicted the prognosis of rectal cancer patients treated with nCRT. Analysis based on the CRTR score suggested that immunotherapy, telomerase inhibitors, aurora kinase A inhibitors, and JAK/STAT pathway inhibitors are potential options for improving the efficacy of nCRT in rectal cancer. KIF14, a key gene within the CRTR model, may serve as a potential predictive biomarker of radiosensitivity in rectal cancer. Our study contributes to the prediction of nCRT treatment response, identifying nCRT-resistant populations to promote personalized precision therapy, and provides a basis for future research.

## Supplementary Information

Below is the link to the electronic supplementary material.

Supplementary Material 1.

Supplementary Material 2.

## Data Availability

All the data generated in this study are included in this published article. R codes for statistical analysis are shown in Supplementary File 1.
