# Supplementary material for: A 13-gene prognostic model developed using machine learning to predict the response to neoadjuvant chemoradiotherapy in rectal carcinoma
Source: Cancer Cell Int. 2026 Mar 21;26:181. doi: 10.1186/s12935-026-04256-9 (PMC13130406; doi:10.1186/s12935-026-04256-9)
Supplement: Supplementary file 1 — Supplementary Material 1. [file 12935_2026_4256_MOESM1_ESM.docx]

**Supplementary File 1**


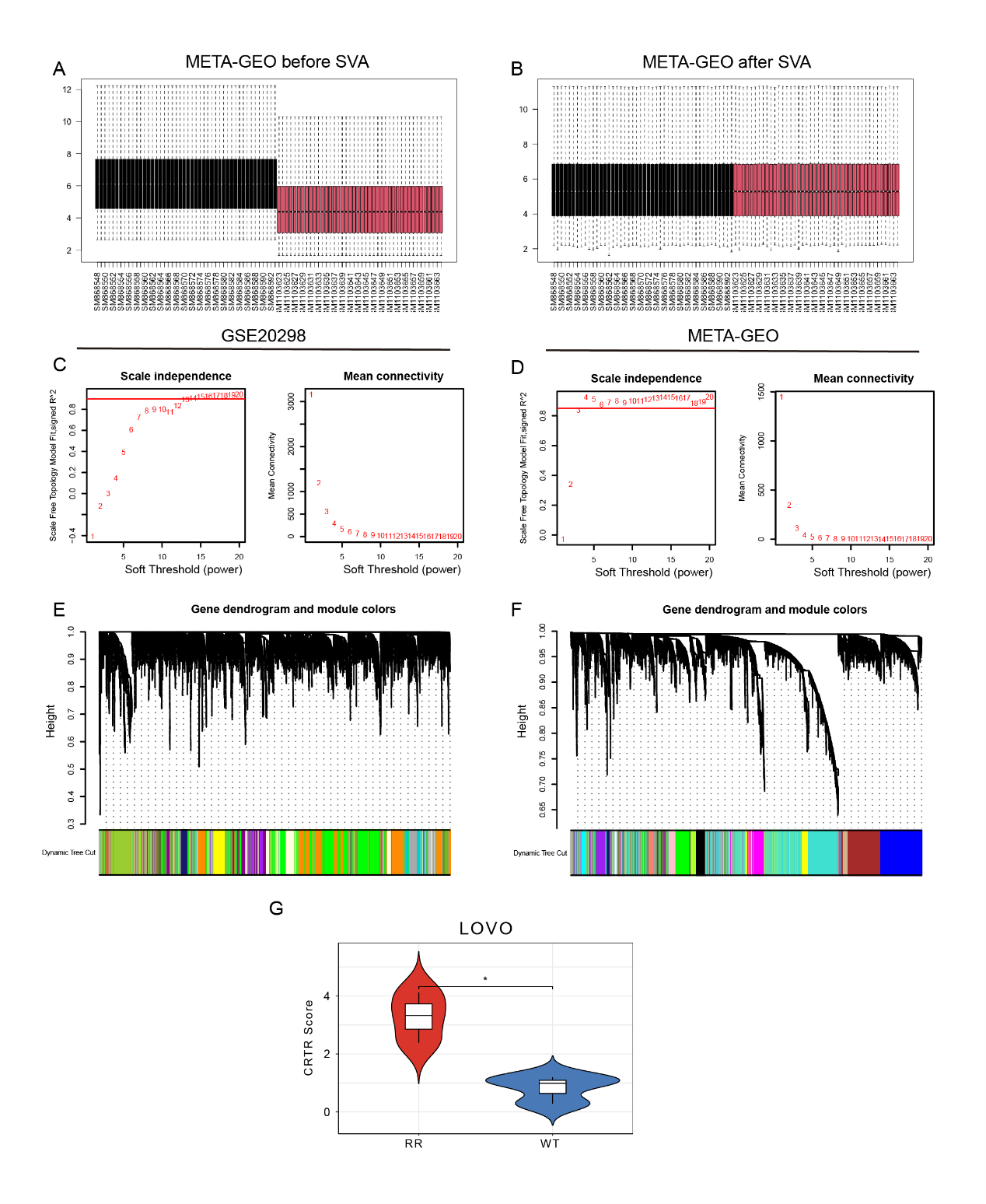


**Supplementary Figure 1. A-B.** Boxplots showing the expression distributions before **(A)** and after **(B)** SVA correction in the META-GEO dataset, illustrating the successful removal of batch effects post-correction. **C-D.** The optimal soft-threshold power. The thresholds of GSE20298 and META GEO are 13 and 3, respectively. **E-F.** Weighted co-expression network modeling based on selected power values. **G.** Distribution of the CRTR score between LOVO radioresistant cell line and LOVO wide type cell line.


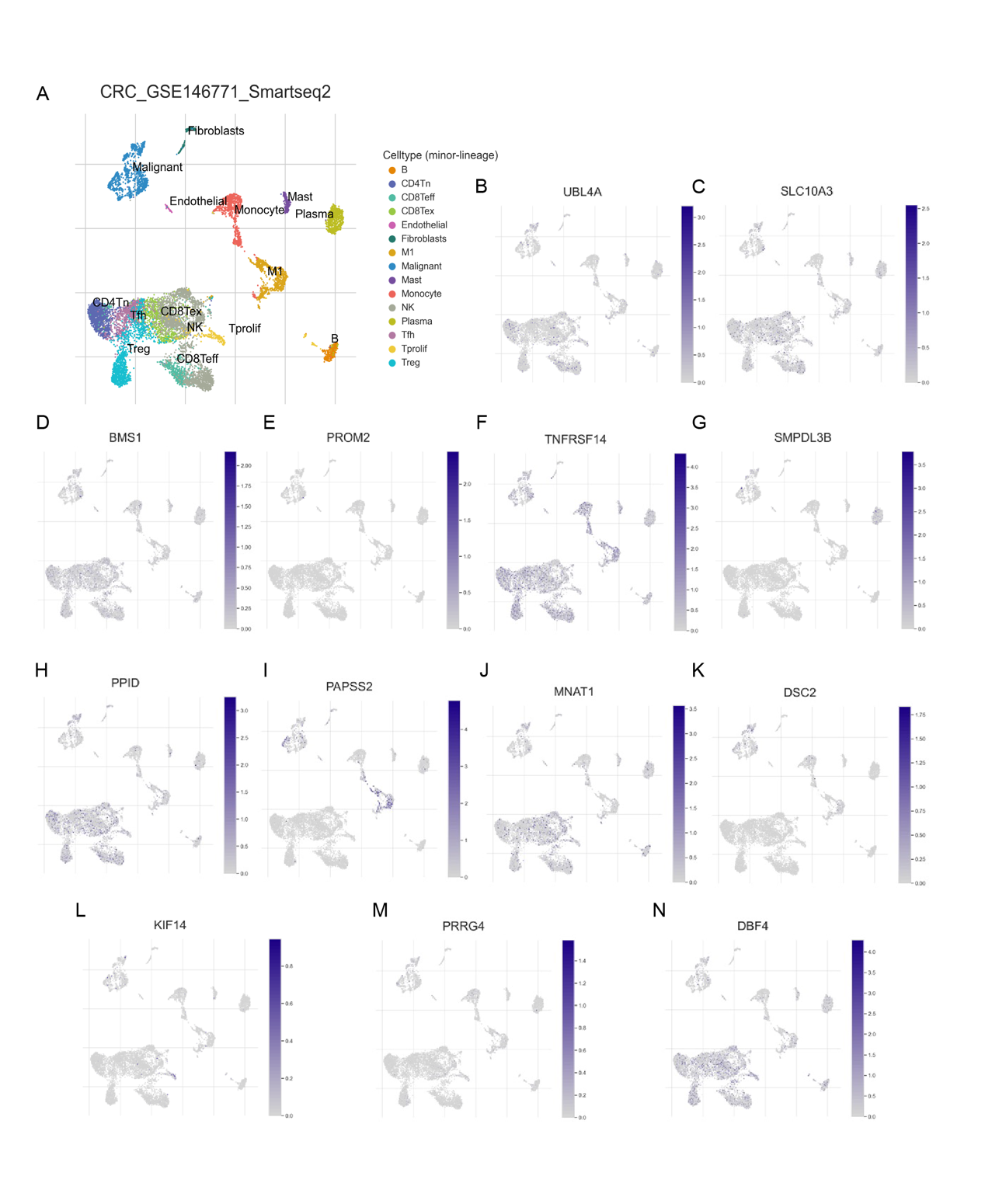


**Supplementary Figure 2. A.** UMAP plot showing distinct cell populations in the GSE146771 dataset, categorized by cell type. **B-N.** Expression patterns of CRTR score genes (KIF14, DBF4, UBL4A, SLC10A3, PRRG4, PAPSS2BMS1, DSC2, PROM2, MNAT1, PPID, SMPDL3B, TNFRSF14) across the cell types, with color intensity indicating expression levels.


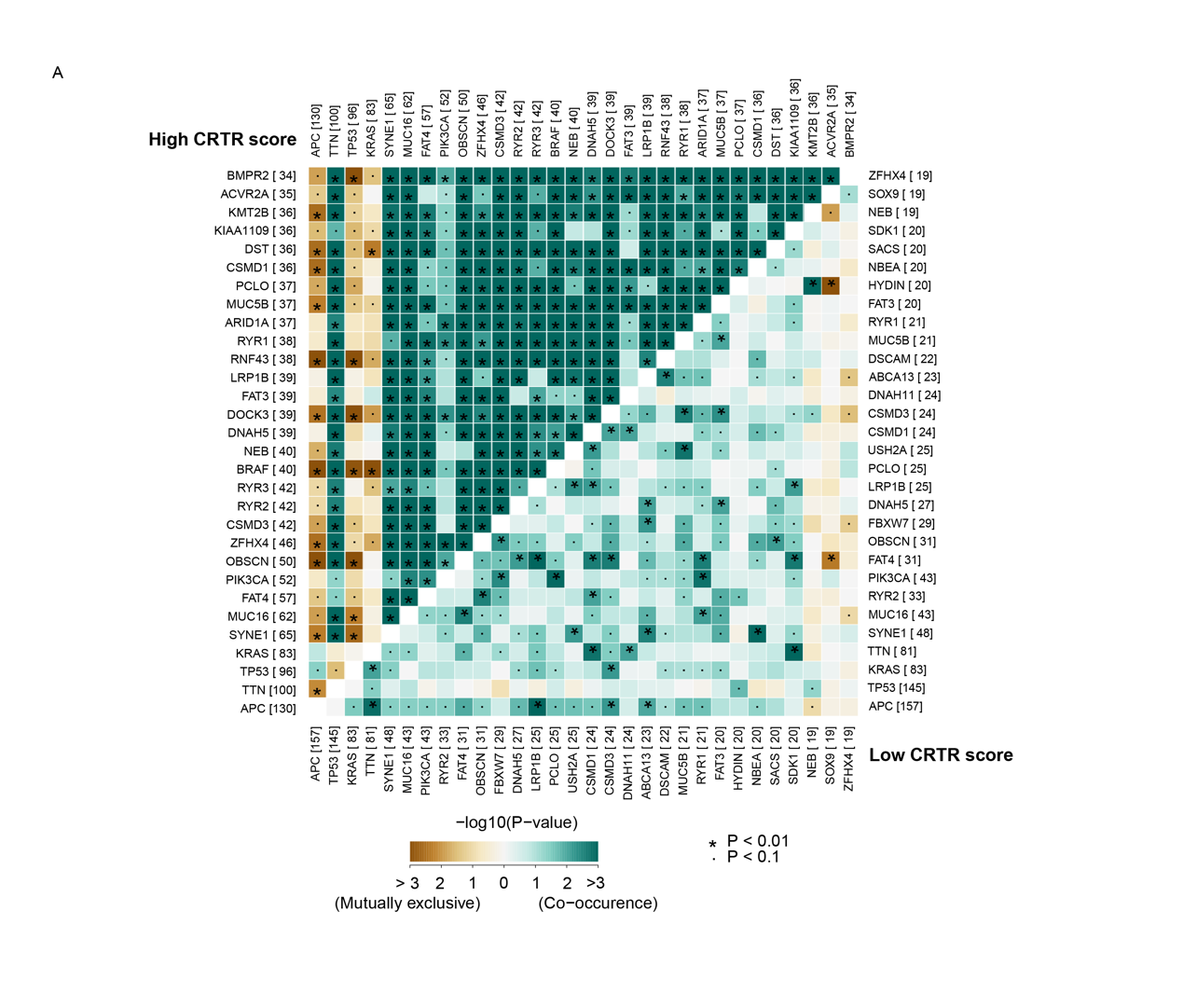


**Supplementary Figure 3. A.** Heatmaps showing the association of co-occurrence and exclusive mutation among the top 30 mutated genes in high and low CRTR score groups.


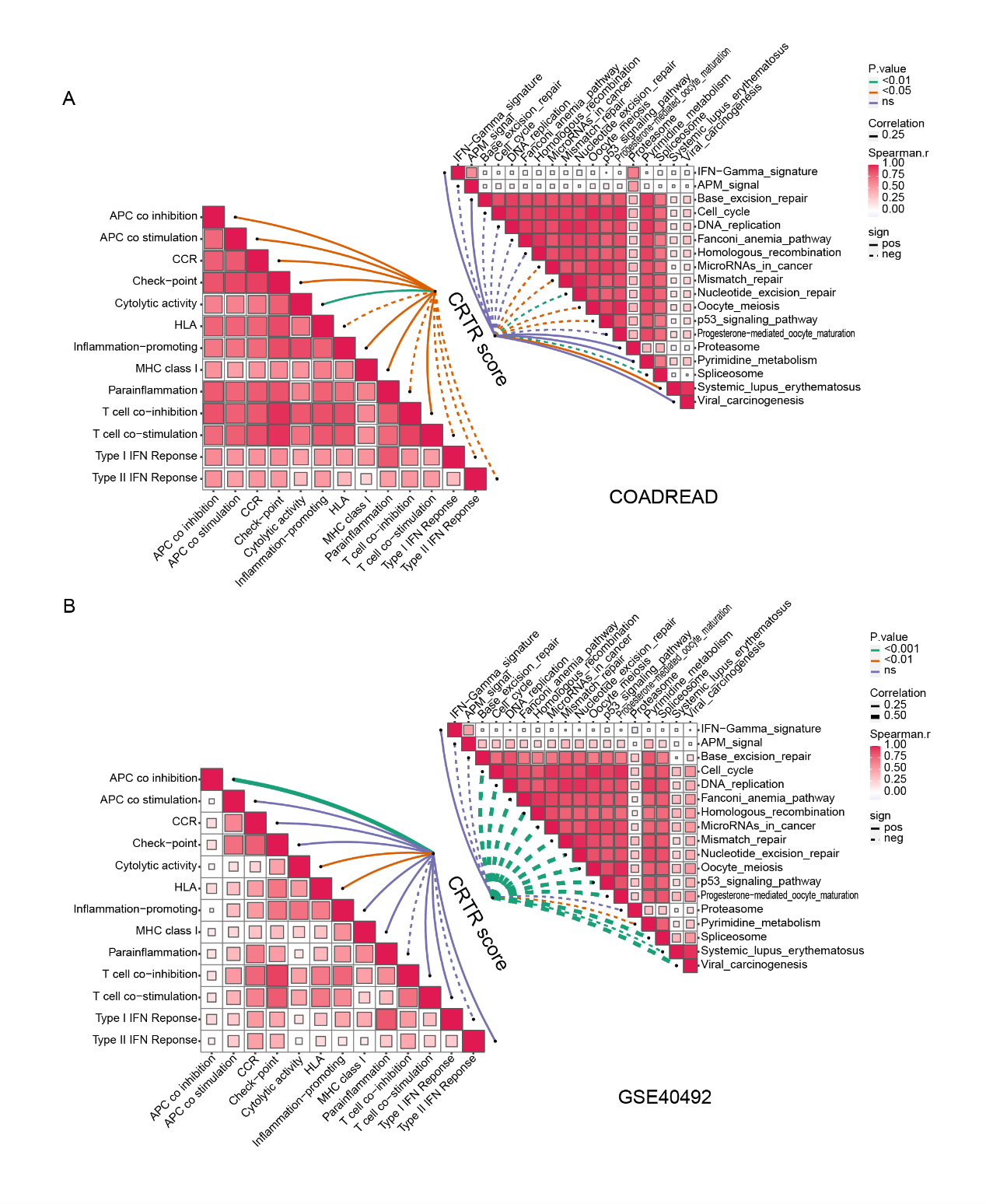


**Supplementary Figure 4. A.** The correlations between CRTR score and both the steps of the cancer immunity cycle and the enrichment scores of immunotherapy-associated pathways in TCGA-COADREAD. **B.** The correlations between CRTR score and both the steps of the cancer immunity cycle and the enrichment scores of immunotherapy-associated pathways in GSE40492.


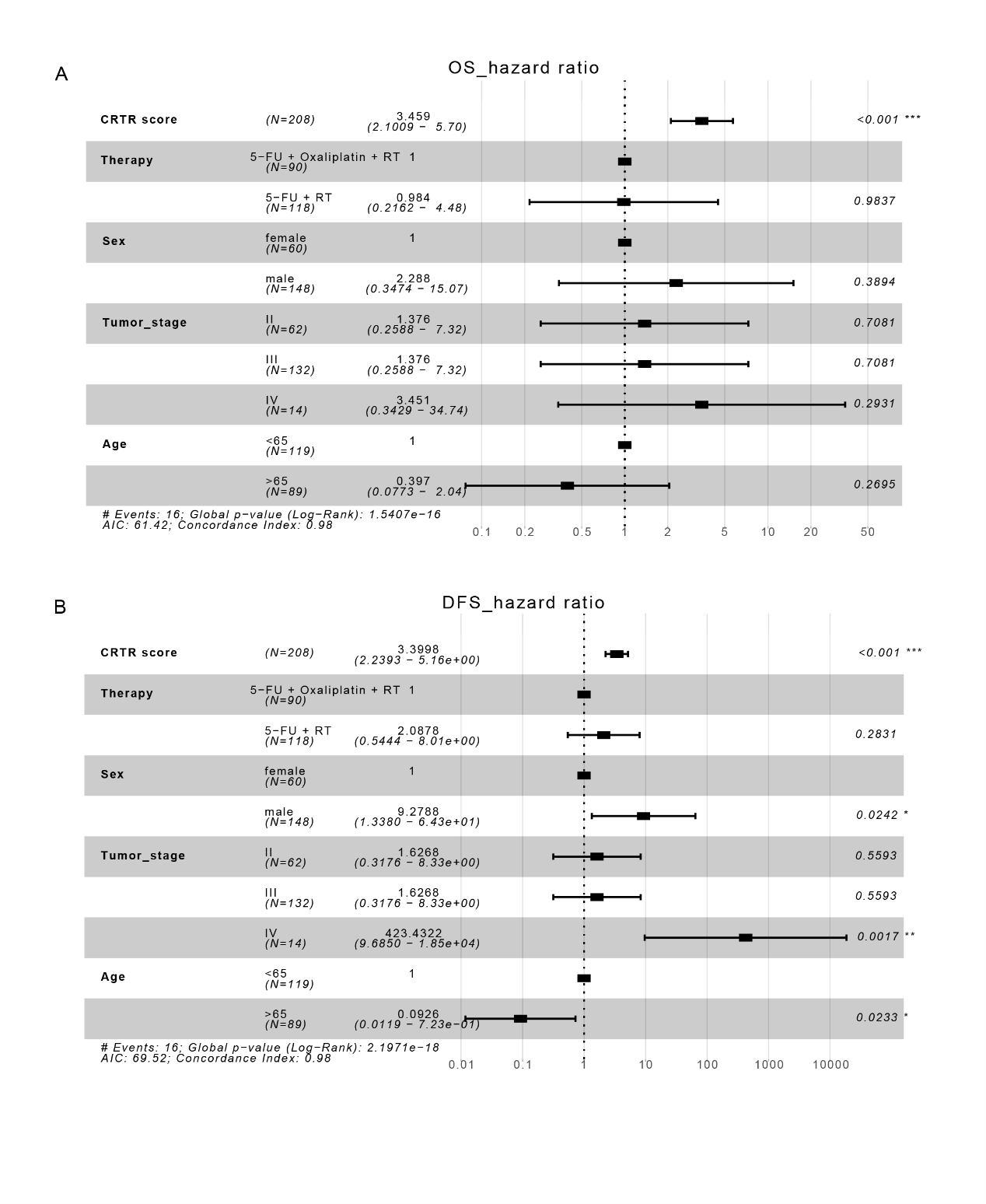


**Supplementary Figure 5.** Multivariate Cox regression analysis of OS (A) and RFS (B) in GSE40492.


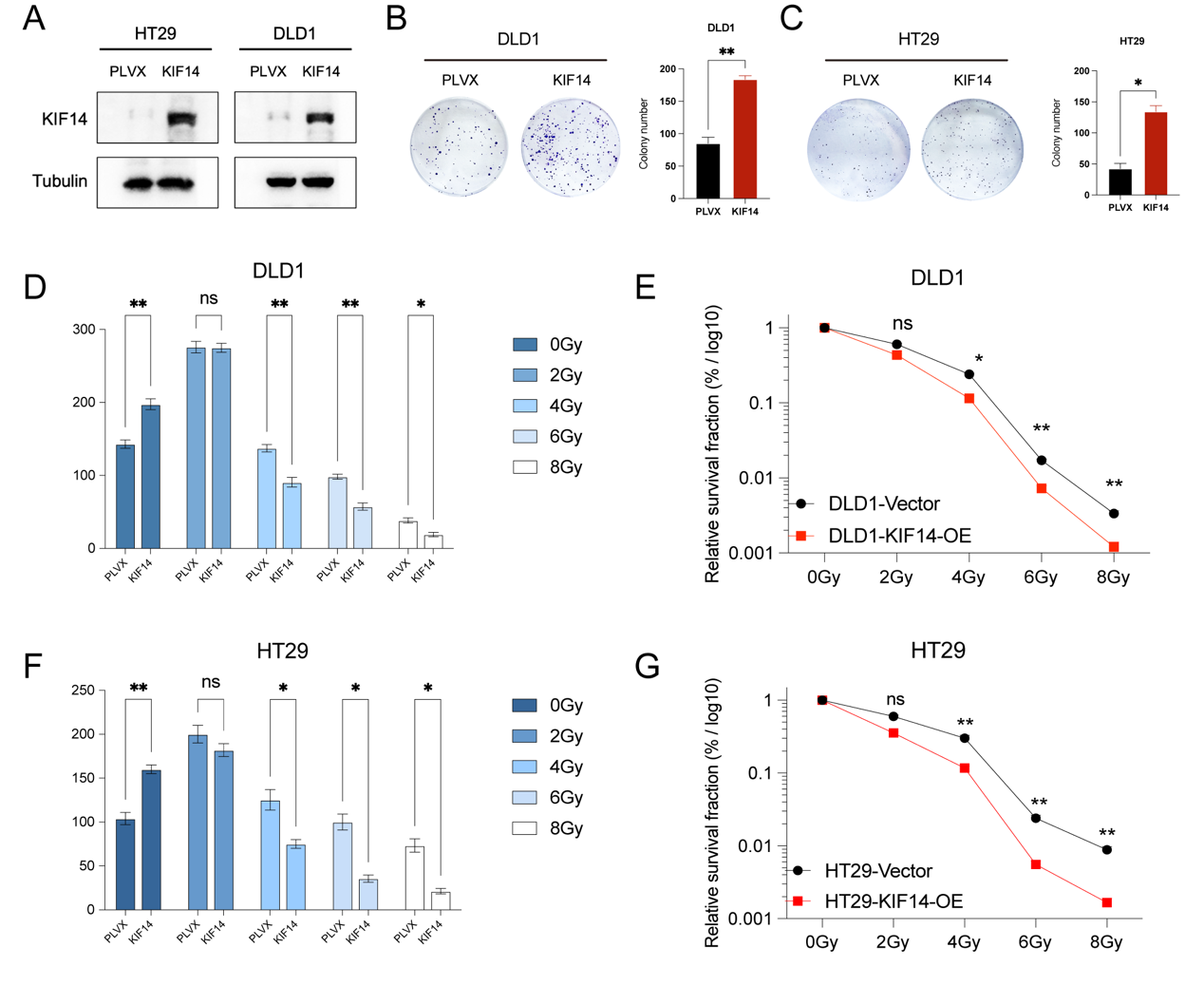


**Supplementary Figure 6. A.** Identification of stably transfected cells overexpressing exogenous KIF14. **B-C.** Plate cloning assay to detect the proliferation of ability of DLD1 and HT29 cells after overexpression of KIF14. **D-G.** DLD1-vector and DLD1-KIF14 cells, as well as HT29-vector and HT29-KIF14 cells were used for clonogenic formation assay, and the results were displayed through the histogram and a linear quadratic model. The experiment was repeated three times independently.
