# Supplementary material for: A 13-gene prognostic model developed using machine learning to predict the response to neoadjuvant chemoradiotherapy in rectal carcinoma
Source: Cancer Cell Int. 2026 Mar 21;26:181. doi: 10.1186/s12935-026-04256-9 (PMC13130406; doi:10.1186/s12935-026-04256-9)
Supplement: Supplementary file 2 — Supplementary Material 2. [file 12935_2026_4256_MOESM2_ESM.pdf]

```
########GSE87211 ########
gset <-    getGEO(GEO='GSE87211', destdir=".",getGPL = F)
gset2 <- gset[["GSE87211_series_matrix.txt.gz"]]
pd <- pData(gset2[1])
raw_dir = "/home/gzh/to_senior.qiu/3.GSE87211&GSE40492/GSE87211_RAW/"
dir(raw_dir)
raw_datas = paste0(raw_dir,"/",dir(raw_dir))
raw_order = str_extract(raw_datas,"GSM\\d*")
pd = pd[match(raw_order,rownames(pd)),]
library(limma)
raw_data <- read.maimages(raw_datas,
                          source="agilent",
                          green.only=TRUE,
                          other.columns = "gIsWellAboveBG")

raw_data$genes$No <- rownames(raw_data$genes)
GPL13497_soft <- getGEO("GPL13497", destdir=".")
GPL13497_soft <- Table(GPL13497_soft)
anno_87211 <- GPL13497_soft
tmp <- anno_87211$GENE_SYMBOL==''
table(tmp)
# tmp
# FALSE    TRUE
# 29833    4351
probe2symbol<-anno_87211[!tmp,c(1,7)]
y <- limma::backgroundCorrect(raw_data, method="normexp")#
Control <- y$genes$ControlType==1L
table(Control)
IsExpr <- rowSums(y$other$gIsWellAboveBG > 0) >= (ncol(y)/2) #n=一半。

table(IsExpr)
table(!Control & IsExpr)
yfilt <- y[!Control & IsExpr,]
dim(yfilt)
exprs <-    yfilt@.Data[[1]] %>% as.data.frame()
colnames(exprs) = str_extract(colnames(exprs),"GSM\\d*")
raw_anno = yfilt$genes
nrow(raw_anno)
nrow(exprs)#36680
exprs$ProbeName <- raw_anno$ProbeName
exprs <- exprs[,c(ncol(exprs),1:ncol(exprs)-1)]
probe2symbol <- distinct(probe2symbol,ID,.keep_all = T)
table(duplicated(probe2symbol$ID))
exprs <- merge(probe2symbol,exprs,by.x=1,by.y=1)
```

```
exprs1 <- aggregate(exprs[,-1], list(exprs$GENE_SYMBOL), FUN = mean) %>%
column_to_rownames(., 'Group.1')
raw_anno_87211 <- raw_anno
exprs_87211 <- exprs
pd_87211 <- pd
save(raw_anno_87211,exprs_87211,pd_87211,file = 'GSE87211_exprs_phe.RData')

########GSE40492 #########
gset <-   getGEO(GEO='GSE40492', destdir=".",getGPL = F)
gset2 <- gset$GSE40492_series_matrix.txt.gz
pd <- pData(gset2[1])
pd_40492 <- pd
raw_dir = '/home/gzh/to_senior.qiu/3.GSE87211&GSE40492/GSE40492_RAW'
dir(raw_dir)
raw_datas = paste0(raw_dir,"/",dir(raw_dir)[-1])
raw_order = str_extract(raw_datas,"GSM\\d*")
pd = pd[match(raw_order,rownames(pd)),]
library(limma)
raw_data <- read.maimages(raw_datas,
                          source="agilent",
                          green.only=TRUE,
                          other.columns = "gIsWellAboveBG")

raw_data$genes$No <- rownames(raw_data$genes)


GPL10332_soft <- getGEO('GPL10332', destdir=".")
GPL10332_soft <- Table(GPL10332_soft)
anno_40492 <- GPL10332_soft
anno <- GPL10332_soft
table(anno$NAME == anno$SPOT_ID)
tmp <- anno$GENE_SYMBOL==''
table(tmp)
y <- limma::backgroundCorrect(raw_data, method="normexp")
Control <- y$genes$ControlType==1L
table(Control)
IsExpr <- rowSums(y$other$gIsWellAboveBG > 0) >= (ncol(y)/2) #n=一半。

table(IsExpr)
table(!Control & IsExpr)
yfilt <- y[!Control & IsExpr,]
dim(yfilt)
yfilt <- normalizeBetweenArrays(yfilt,method = "quantile")
exprs <-   yfilt@.Data[[1]] %>% as.data.frame()
```

```
raw_anno = yfilt$genes
nrow(raw_anno)
nrow(exprs)#35924
exprs$ProbeName <- raw_anno$ProbeName
exprs <- exprs[,c(ncol(exprs),1:ncol(exprs)-1)]
probe2symbol <- distinct(probe2symbol,NAME,.keep_all = T)
table(duplicated(probe2symbol$NAME))
exprs <- merge(probe2symbol,exprs,by.x=1,by.y=1)
exprs <- exprs[,-1]

exprs1 <- aggregate(exprs[,-1], list(exprs$GENE_SYMBOL), FUN = mean) %>%
column_to_rownames(., 'Group.1')
exprs <- exprs1
exprs[1:4,1:4]
raw_anno_40492 <- raw_anno
exprs_40492 <- exprs
pd_40492 <- pd
CairoPNG('boxplot_87211_z.png', width = 1000, height = 600)
boxplot(exprs_87211,outline=FALSE, notch=T, las=2)
dev.off()
CairoPNG('boxplot_40492_z.png', width = 1000, height = 600)
boxplot(exprs_40492,outline=FALSE, notch=T, las=2)
dev.off()

########WGCNA #########
expFile <- "GSE20298toWGCNA.csv"
pheFile <- "GSE20298_phe.csv"
exp=read.csv(expFile, header=T) %>% column_to_rownames(.,'X')
pd <- read.csv(pheFile, header=T) %>% column_to_rownames(.,'X')
exp=as.matrix(exp)
dimnames=list(rownames(exp),colnames(exp))
data=matrix(as.numeric(as.matrix(exp)),nrow=nrow(exp),dimnames=dimnames)
data=data[apply(data,1,sd)>0.6,]
dim(data)
datExpr0=t(data)
pdf(file = "1_sample_cluster.pdf", width = 12, height = 9)
par(cex = 0.6)
par(mar = c(0,4,2,0))
plot(sampleTree, main = "Sample clustering to detect outliers", sub="", xlab="",
cex.lab = 1.5, cex.axis = 1.5, cex.main = 2)
abline(h = 140, col = "red")
dev.off()
clust = cutreeStatic(sampleTree, cutHeight = 140, minSize = 10)
table(clust)
```

```
keepSamples = (clust==1)
datExpr0 = datExpr0[keepSamples, ]
geoSamples = rownames(datExpr0)
traitSamples =pd[which(pd$characteristics_ch1.2!='sf2: NA'),1]
sameSample=intersect(geoSamples,traitSamples)
datExpr0=datExpr0[sameSample,]
rownames(pd) <- pd$title
datTraits=pd[sameSample,c(1,40)]
datTraits <- data.frame(datTraits$title,datTraits$sf2.ch1)
datTraits <- column_to_rownames(datTraits,'datTraits.title')
colnames(datTraits) <- 'sf2'
sampleTree2 = hclust(dist(datExpr0), method = "average")
traitColors = numbers2colors(datTraits, signed = FALSE)
pdf(file="2_sample_heatmap.pdf",width=12,height=12)
plotDendroAndColors(sampleTree2, traitColors,
                    groupLabels = names(datTraits),
                    main = "Sample dendrogram and trait heatmap")
dev.off()
enableWGCNAThreads()
powers = c(1:20)
sft = pickSoftThreshold(datExpr0, powerVector = powers, verbose = 5)
pdf(file="3_scale_independence.pdf",width=9,height=5)
par(mfrow = c(1,2))
cex1 = 0.9
plot(sft$fitIndices[,1], -sign(sft$fitIndices[,3])*sft$fitIndices[,2],
     xlab="Soft Threshold (power)",ylab="Scale Free Topology Model Fit,signed
R^2",type="n",
     main = paste("Scale independence"));
text(sft$fitIndices[,1], -sign(sft$fitIndices[,3])*sft$fitIndices[,2],
     labels=powers,cex=cex1,col="red");
abline(h=0.90,col="red")
plot(sft$fitIndices[,1], sft$fitIndices[,5],
     xlab="Soft Threshold (power)",ylab="Mean Connectivity", type="n",
     main = paste("Mean connectivity"))
text(sft$fitIndices[,1], sft$fitIndices[,5], labels=powers, cex=cex1,col="red")
dev.off()
softPower =sft$powerEstimate
adjacency = adjacency(datExpr0, power = softPower)
softPower
TOM = TOMsimilarity(adjacency)
dissTOM = 1-TOM
geneTree = hclust(as.dist(dissTOM), method = "average");
pdf(file="4_gene_clustering.pdf",width=12,height=9)
plot(geneTree, xlab="", sub="", main = "Gene clustering on TOM-based
```

```
dissimilarity",
        labels = FALSE, hang = 0.04)
dev.off()
minModuleSize = 60
dynamicMods = cutreeDynamic(dendro = geneTree, distM = dissTOM,
                            deepSplit = 2, pamRespectsDendro = FALSE,
                            minClusterSize = minModuleSize);
table(dynamicMods)
dynamicColors = labels2colors(dynamicMods)
table(dynamicColors)
pdf(file="5_Dynamic_Tree.pdf",width=8,height=6)
plotDendroAndColors(geneTree, dynamicColors, "Dynamic Tree Cut",
                    dendroLabels = FALSE, hang = 0.03,
                    addGuide = TRUE, guideHang = 0.05,
                    main = "Gene dendrogram and module colors")
dev.off()
MEList = moduleEigengenes(datExpr0, colors = dynamicColors)
MEs = MEList$eigengenes
MEDiss = 1-cor(MEs);
METree = hclust(as.dist(MEDiss), method = "average")
pdf(file="6_Clustering_module.pdf",width=7,height=6)
plot(METree, main = "Clustering of module eigengenes",
        xlab = "", sub = "")
MEDissThres = 0.3
abline(h=MEDissThres, col = "red")
dev.off()
merge = mergeCloseModules(datExpr0, dynamicColors, cutHeight = MEDissThres,
verbose = 3)
mergedColors = merge$colors
mergedMEs = merge$newMEs
pdf(file="7_merged_dynamic.pdf", width = 9, height = 6)
plotDendroAndColors(geneTree, mergedColors,"Dynamic Tree Cut",
                    dendroLabels = FALSE, hang = 0.03,
                    addGuide = TRUE, guideHang = 0.05,
                    main = "Gene dendrogram and module colors")
dev.off()
moduleColors = mergedColors
table(moduleColors)
colorOrder = c("grey", standardColors(50))
moduleLabels = match(moduleColors, colorOrder)-1
MEs = mergedMEs
nGenes = ncol(datExpr0)
nSamples = nrow(datExpr0)
moduleTraitCor = cor(MEs, datTraits, use = "p")
```

```
moduleTraitPvalue = corPvalueStudent(moduleTraitCor, nSamples)
pdf(file="8_Module_trait.pdf", width=6, height=5.5)
textMatrix = paste(signif(moduleTraitCor, 2), "\n(",
                   signif(moduleTraitPvalue, 1), ")", sep = "")
dim(textMatrix) = dim(moduleTraitCor)
par(mar = c(5, 10, 3, 3))
labeledHeatmap(Matrix = moduleTraitCor,
               xLabels = 'sf2',#names(datTraits)
               yLabels = names(MEs),
               ySymbols = names(MEs),
               colorLabels = FALSE,
               colors = blueWhiteRed(50),
               textMatrix = textMatrix,
               setStdMargins = FALSE,
               cex.text = 0.5,
               zlim = c(-1,1),
               main = paste("Module-trait relationships"))
dev.off()

#machine learning#####
# 1.1    CoxBoost#####
set.seed(seed)
pen <- optimCoxBoostPenalty(est_dd[,'OS.time'],est_dd[,'OS'],as.matrix(est_dd[,-
c(1,2)]),
                            trace=TRUE,start.penalty=500,parallel = T)
cv.res <- cv.CoxBoost(est_dd[,'OS.time'],est_dd[,'OS'],as.matrix(est_dd[,-c(1,2)]),

maxstepno=500,K=10,type="verweij",penalty=pen$penalty)
fit <- CoxBoost(est_dd[,'OS.time'],est_dd[,'OS'],as.matrix(est_dd[,-c(1,2)]),
                stepno=cv.res$optimal.step,penalty=pen$penalty)
rs <-
lapply(val_dd_list,function(x){cbind(x[,1:2],RS=as.numeric(predict(fit,newdata=x[,-
c(1,2)], newtime=x[,1], newstatus=x[,2], type="lp")))})
cc <-
data.frame(Cindex=sapply(rs,function(x){as.numeric(summary(coxph(Surv(OS.time,
OS)~RS,x))$concordance[1])}))%>%
  rownames_to_column('ID')
cc$Model <- paste0('CoxBoost')
result <- rbind(result,cc)

# 2  Enet #####
x1 <- as.matrix(est_dd[,pre_var])
x2 <- as.matrix(Surv(est_dd$OS.time,est_dd$OS))
for (alpha in seq(0.1,0.9,0.1)) {
```

```
  set.seed(seed)
  fit = cv.glmnet(x1, x2,family = "cox",alpha=alpha,nfolds = 10)
  rs <-
lapply(val_dd_list,function(x){cbind(x[,1:2],RS=as.numeric(predict(fit,type='link',ne
wx=as.matrix(x[,-c(1,2)]),s=fit$lambda.min)))})
  cc <-
data.frame(Cindex=sapply(rs,function(x){as.numeric(summary(coxph(Surv(OS.time,
OS)~RS,x))$concordance[1])}))%>%
    rownames_to_column('ID')
  cc$Model <- paste0('Enet','[α=',alpha,']')
  result <- rbind(result,cc)
}
# 3 GBM#####
set.seed(seed)
fit <- gbm(formula = Surv(OS.time,OS)~.,data = est_dd,distribution = 'coxph',
           n.trees = 10000,
           interaction.depth = 3,
           n.minobsinnode = 10,
           shrinkage = 0.001,
           cv.folds = 10,n.cores = 6)
# find index for number trees with minimum CV error
best <- which.min(fit$cv.error)
set.seed(seed)
fit <- gbm(formula = Surv(OS.time,OS)~.,data = est_dd,distribution = 'coxph',
           n.trees = best,
           interaction.depth = 3,
           n.minobsinnode = 10,
           shrinkage = 0.001,
           cv.folds = 10,n.cores = 8)
rs <- lapply(val_dd_list,function(x){cbind(x[,1:2],RS=as.numeric(predict(fit,x,n.trees
= best,type = 'link')))})
cc <-
data.frame(Cindex=sapply(rs,function(x){as.numeric(summary(coxph(Surv(OS.time,
OS)~RS,x))$concordance[1])}))%>%
  rownames_to_column('ID')
cc$Model <- paste0('GBM')
result <- rbind(result,cc)
# 4.1   Lasso#####
x1 <- as.matrix(est_dd[,pre_var])
x2 <- as.matrix(Surv(est_dd$OS.time,est_dd$OS))
set.seed(seed)
fit = cv.glmnet(x1, x2,family = "cox",alpha=1,nfolds = 10)
rs <-
lapply(val_dd_list,function(x){cbind(x[,1:2],RS=as.numeric(predict(fit,type='link',ne
```

```
wx=as.matrix(x[,-c(1,2)]),s=fit$lambda.min)))})
cc <-
data.frame(Cindex=sapply(rs,function(x){as.numeric(summary(coxph(Surv(OS.time,
OS)~RS,x))$concordance[1])}))%>%
  rownames_to_column('ID')
cc$Model <- paste0('Lasso')
result <- rbind(result,cc)

# 5 plsRcox#####
set.seed(seed)
cv.plsRcox.res=cv.plsRcox(list(x=est_dd[,pre_var],time=est_dd$OS.time,status=est_d
d$OS),nt=10,verbose = FALSE)
fit <-
plsRcox(est_dd[,pre_var],time=est_dd$OS.time,event=est_dd$OS,nt=as.numeric(cv.p
lsRcox.res[5]))
rs <-
lapply(val_dd_list,function(x){cbind(x[,1:2],RS=as.numeric(predict(fit,type="lp",new
data=x[,-c(1,2)])))})
cc <-
data.frame(Cindex=sapply(rs,function(x){as.numeric(summary(coxph(Surv(OS.time,
OS)~RS,x))$concordance[1])}))%>%
  rownames_to_column('ID')
cc$Model <- paste0('plsRcox')
result <- rbind(result,cc)

# 6 Ridge#####
x1 <- as.matrix(est_dd[,pre_var])
x2 <- as.matrix(Surv(est_dd$OS.time,est_dd$OS))
set.seed(seed)
fit = cv.glmnet(x1, x2,family = "cox",alpha=0,nfolds = 10)
rs <-
lapply(val_dd_list,function(x){cbind(x[,1:2],RS=as.numeric(predict(fit,type='link',ne
wx=as.matrix(x[,-c(1,2)]),s=fit$lambda.min)))})
cc <-
data.frame(Cindex=sapply(rs,function(x){as.numeric(summary(coxph(Surv(OS.time,
OS)~RS,x))$concordance[1])}))%>%
  rownames_to_column('ID')
cc$Model <- paste0('Ridge')
result <- rbind(result,cc)

# 7.1     RSF#####
set.seed(seed)
fit <- rfsrc(Surv(OS.time,OS)~.,data = est_dd,
                  ntree = 10,nodesize = 10,
```

```
                   splitrule = 'logrank',
                   importance = T,
                   proximity = T,
                   forest = T,
                   seed = seed)
rs <- lapply(val_dd_list,function(x){cbind(x[,1:2],RS=predict(fit,newdata =
x)$predicted)})
cc <-
data.frame(Cindex=sapply(rs,function(x){as.numeric(summary(coxph(Surv(OS.time,
OS)~RS,x))$concordance[1])}))%>%
  rownames_to_column('ID')
cc$Model <- 'RSF'
result <- rbind(result,cc)

# 8.1      StepCox #####
for (direction in c("both", "backward", "forward")) {
  fit <- step(coxph(Surv(OS.time,OS)~.,est_dd),direction = direction)
  rs <- lapply(val_dd_list,function(x){cbind(x[,1:2],RS=predict(fit,type =
'risk',newdata = x))})
  cc <-
data.frame(Cindex=sapply(rs,function(x){as.numeric(summary(coxph(Surv(OS.time,
OS)~RS,x))$concordance[1])}))%>%
    rownames_to_column('ID')
  cc$Model <- paste0('StepCox','[',direction,']')
  result <- rbind(result,cc)
}

# 9 SuperPC######
data <- list(x=t(est_dd[,-
c(1,2)]),y=est_dd$OS.time,censoring.status=est_dd$OS,featurenames=colnames(est_
dd)[-c(1,2)])
set.seed(seed)
fit <- superpc.train(data = data,type = 'survival',s0.perc = 0.5) #default
cv.fit <- superpc.cv(fit,data,n.threshold = 20,#default
                     n.fold = 10,
                     n.components=3,
                     min.features=5,
                     max.features=nrow(data$x),
                     compute.fullcv= TRUE,
                     compute.preval=TRUE)
rs <- lapply(val_dd_list,function(w){
  test <- list(x=t(w[,-
c(1,2)]),y=w$OS.time,censoring.status=w$OS,featurenames=colnames(w)[-c(1,2)])
  ff <- superpc.predict(fit,data,test,threshold =
```

```
cv.fit$thresholds[which.max(cv.fit[["scor"]][1,])],n.components = 1)
   rr <- as.numeric(ff$v.pred)
   rr2 <- cbind(w[,1:2],RS=rr)
   return(rr2)
})
cc <-
data.frame(Cindex=sapply(rs,function(x){as.numeric(summary(coxph(Surv(OS.time,
OS)~RS,x))$concordance[1])}))%>%
   rownames_to_column('ID')
cc$Model <- paste0('SuperPC')
result <- rbind(result,cc)

# 10 survival-SVM#####
fit = survivalsvm(Surv(OS.time,OS)~., data= est_dd, gamma.mu = 1)
rs <- lapply(val_dd_list,function(x){cbind(x[,1:2],RS=as.numeric(predict(fit,
x)$predicted))})
cc <-
data.frame(Cindex=sapply(rs,function(x){as.numeric(summary(coxph(Surv(OS.time,
OS)~RS,x))$concordance[1])}))%>%
   rownames_to_column('ID')
cc$Model <- paste0('survival-SVM')
result <- rbind(result,cc)

result2 <- result
result2$Model <- gsub('α','a',result2$Model)
range(result2$Cindex)

CairoPNG(filename = 'Cindex.png',width = 600,height = 1200,dip=300)
result2%>%#filter(ID!='Training_Dataset')%>%
   ggplot(aes(Cindex,reorder(Model,Cindex)))+
   geom_bar(width = 0.7,stat = 'summary',fun='mean',fill='orange2')+
   theme_classic()+
   labs(y=NULL)
dev.off()
dd <- result2%>%
   #filter(ID!='Training_Dataset')%>%
   group_by(Model)%>%
   summarise(Cindex=mean(Cindex))
dd2 <- pivot_wider(result2,names_from = 'ID',values_from =
'Cindex')%>%as.data.frame()
CairoPNG(filename = 'Cindex.png',width = 600,height = 1200,dip=300)
result3%>%#filter(ID!='Training_Dataset')%>%
   ggplot(aes(Cindex,reorder(Model,Cindex)))+
   geom_bar(width = 0.7,stat = 'summary',fun='mean',fill='orange2')+
```

```
  theme_classic()+
  labs(y=NULL)
dev.off()
################heatmap######
library(RColorBrewer)
library(ComplexHeatmap)
Cindex_mat <- dd2 %>% column_to_rownames(.,'Model')
avg_Cindex <- apply(Cindex_mat, 1, mean)
avg_Cindex <- sort(avg_Cindex, decreasing = T)
Cindex_mat <- Cindex_mat[names(avg_Cindex), ]
avg_Cindex <- as.numeric(format(avg_Cindex, digits = 3, nsmall = 3))
row_ha = rowAnnotation(bar = anno_barplot(avg_Cindex, bar_width = 0.8, border =
FALSE,
                                          gp = gpar(fill = "steelblue", col =
NA),
                                          add_numbers = T,
numbers_offset = unit(-10, "mm"),
                                          axis_param = list("labels_rot" =
0),
                                          numbers_gp = gpar(fontsize = 9,
col = "white"),
                                          width = unit(3, "cm")),
                      show_annotation_name = F)

CohortCol <- brewer.pal(n = ncol(Cindex_mat), name = "Paired")
#CohortCol <- c("#92B7D5","#F3B879")
names(CohortCol) <- colnames(Cindex_mat)
col_ha = columnAnnotation("Cohort" = colnames(Cindex_mat),
                          col = list("Cohort" = CohortCol),
                          show_annotation_name = F)
cellwidth = 2.5
cellheight = 0.5

###########UnimultiCox############
library(tidyverse)
surv_expr <-rbind(Training_Dataset,Testing_Dataset1)
surv_expr <- cbind(surv_expr[,1:2],surv_expr[,rid])
colnames(surv_expr)[1:2] <- c("time","status")
FUN_surv <- function(i, surv_expr) {
  Sur <- Surv(surv_expr$time, as.numeric(surv_expr$status))
  cox <- coxph(Sur ~ surv_expr[, i], data = surv_expr)
  coxSummary <- summary(cox)
  gene <- names(surv_expr)[i]
  HR <- coxSummary$coefficients[, "exp(coef)"]
```

```
   lower <- coxSummary$conf.int[, 3]
   upper <- coxSummary$conf.int[, 4]
   PValue <- round(coxSummary$coefficients[, 5], 6)
   res <- data.frame(gene = gene, HR = HR, lower.95 = lower, upper.95 = upper,
pvalue = PValue)
   return(res)
}
l <- pbapply::pblapply(3:ncol(surv_expr), FUN = FUN_surv, surv_expr = surv_expr)
Univar <- do.call(rbind, l)
Univar$gene <- Univar$gene %>% str_replace_all("_", "-")
Univar <- Univar[order(Univar$pvalue), ]

#######nomogram #########
dd <- datadist(multidata)
options(datadist = "dd")
res.cph <- cph(Surv(OS.time,OS) ~ RS + Therapy + Sex + Tumor_stage + Age,
                      data = multidata, x = T, y = T, surv = T,max.iter = 1000)
dfs.cph <- cph(Surv(DFS.time,OS) ~ RS + Therapy + Sex + Tumor_stage +Age,
                      data = multidata, x = T, y = T, surv = T,max.iter = 1000)
surv <- Survival(res.cph)
surv <- Survival(dfs.cph)
surv1 <- function(x)surv(12*1,x)
surv2 <- function(x)surv(12*3,x)
surv3 <- function(x)surv(12*5,x)
surv4 <- function(x)surv(12*10,x)
nom <- nomogram(res.cph,fun = list(surv1, surv2, surv3,surv4 ),
                       fun.at = c(0.05,seq(0.1,0.9,by=0.2),0.95),
                       maxscale=100,funlabel =c( '1 year survival','3 year survival','5
year survival','10 year survival'))
nom <- nomogram(dfs.cph,fun = list(surv1, surv2, surv3,surv4 ),
                       fun.at = c(0.05,seq(0.1,0.9,by=0.2),0.95),
                       maxscale=100,funlabel =c( '1 year survival','3 year survival','5
year survival','10 year survival'))

#########somatic mutation########
files <- list.files(pattern = '*.wxs.aliquot_ensemble_maske',recursive = TRUE)
all_mut <- data.frame()
for (file in files) {
   mut <- read.delim(file,skip = 7, header = T, fill = TRUE,sep = "\t")
   all_mut <- rbind(all_mut,mut)
}
result_vector <- substring(all_mut$Tumor_Sample_Barcode, 1, 15)
all_mut$Tumor_Sample_Barcode <- result_vector
tmp <- unique(all_mut$Tumor_Sample_Barcode)
```

```
tmp2 <- intersect(tmp,rownames(rs_coadread))
a <- all_mut@data %>%
   .[,c("Hugo_Symbol","Variant_Classification","Tumor_Sample_Barcode")] %>%
   as.data.frame() %>%
   mutate(Tumor_Sample_Barcode = substring(.$Tumor_Sample_Barcode,1,12))
gene <- as.character(unique(a$Hugo_Symbol))
sample <- as.character(unique(a$Tumor_Sample_Barcode))
mat <- as.data.frame(matrix("",length(gene),length(sample),
                                        dimnames = list(gene,sample)))
mat_0_1 <- as.data.frame(matrix(0,length(gene),length(sample),
                                        dimnames = list(gene,sample)))
for (i in 1:nrow(a)){
   mat[as.character(a[i,1]),as.character(a[i,3])] <- as.character(a[i,2])
}
for (i in 1:nrow(a)){
   mat_0_1[as.character(a[i,1]),as.character(a[i,3])] <- 1
}
gene_count <- data.frame(gene=rownames(mat_0_1),
                                    count=as.numeric(apply(mat_0_1,1,sum))) %>%
   arrange(desc(count))
gene_top <- gene_count$gene[1:20]
save(mat,mat_0_1,file = "TMB.rda")
write.csv(mat,"all_mut_type.csv")
write.csv(mat_0_1,"all_mut_01.csv")

col = RColorBrewer::brewer.pal(n = 10, name = 'Paired')
names(col) = c('Frame_Shift_Del','Missense_Mutation', 'Nonsense_Mutation',
'Frame_Shift_Ins','In_Frame_Ins',
                      'Splice_Site',
'In_Frame_Del','Nonstop_Mutation','Translation_Start_Site','Multi_Hit')
oncoplot(maf = all_mut1,
             top = 20,
             colors = col,
             fontSize = 0.6,
             clinicalFeatures = c("group"),
             sortByAnnotation = TRUE)
col = RColorBrewer::brewer.pal(n = 10, name = 'Paired')
names(col) = c('Frame_Shift_Del','Missense_Mutation', 'Nonsense_Mutation',
'Frame_Shift_Ins','In_Frame_Ins',
                      'Splice_Site',
'In_Frame_Del','Nonstop_Mutation','Translation_Start_Site','Multi_Hit')
oncoplot(maf = all_mut1,
             genes = result$results$Hugo_Symbol[1:20],
             colors = col,
```

```
                fontSize = 0.6,
                clinicalFeatures = c("group"),
                showMutationNone = TRUE,
                sortByAnnotation = TRUE)

myMut <- all_mut1@variants.per.sample
head(myMut)
myGroup <- rs_coadread1[tmp2,]
head(myGroup)

### TMB#####
TMB_per_sample <- myMut
TMB_per_sample$TMB <- myMut$Variants%/%35
TMB_RS <- merge(TMB_per_sample, myGroup, by="Tumor_Sample_Barcode")
TMB_RS$logTMB <- log(TMB_RS$TMB+1)
head(TMB_RS)
#TMB_clinical_mRNA$Expr <- as.numeric(TMB_clinical_mRNA$Expr)
res <- wilcox.test(TMB ~ group, data = TMB_RS,
                   paired = FALSE, #whether you want a paired test
                   exact = FALSE)
(pvalue <- res$p.value)
### explainer#########
library(survex)
library(survival)
library(randomForestSRC)
rsf_model <- rfsrc(
Surv(time,
status)~.,
data = veteran)
explainer <- explain(rsf_model)
```
